# Supplementary material for: The role of teams in shaping quality of obstetrical care: a cross-sectional study in Dire Dawa, Ethiopia
Source: BMJ Open. 2022 Oct 3;12(10):e066111. doi: 10.1136/bmjopen-2022-066111 (PMC9535209; doi:10.1136/bmjopen-2022-066111)

Appendix 1. Examples of provider arrangements during labor and delivery

Number of providers	First exam	First stage of labor	Delivery care	Immediate newborn care	Immediate maternal postpartum care	Frequency
1						45%
2						38%
3						13%
4						3%
5						0.5%

	Legend
	Provider 1
	Provider 2
	Provider 3
	Provider 4
	Provider 5

Appendix 2. Quality of intrapartum and immediate postpartum care processes with no time constraints on actions

	Percent completed	N
Average of first exam actions	47%	823
Checks woman's HIV status	68%	809
Asks whether woman has experienced headaches or blurred vision	6%	823
Asks whether woman has experienced vaginal bleeding	7%	823
Takes blood pressure during initial client assessment	73%	822
Takes pulse during initial client assessment	67%	822
Washes hands before initial examination	13%	823
Wears gloves before vaginal examination	99%	781
Average of first stage of labor actions	48%	795
At least once, explains what will happen in labor	42%	824
Prepares uterotonic drug to use for AMTSL	81%	791
Uses partograph during labor	53%	793
Prepares bags and masks for neonatal resuscitation	14%	783
Average of third stage of labor actions	88%	828
Correctly administers uterotonic (no time requirement)	93%	820
Assesses completeness of placenta and membranes	74%	819
Assesses for perineal and vaginal membranes	90%	819
Ties or clamps cord	99%	828
Average of immediate newborn care	81%	694
Immediately dries baby with towel	98%	694
Places newborn on mother's abdomen skin-to-skin	64%	690
Average of immediate maternal postpartum stage actions	37%	824
Takes mother's vital signs after birth	3%	823
Palpates uterus after birth	72%	824
Assists mother to initiate breastfeeding	37%	695

Alternate Quality of Intrapartum and Immediate Postpartum Care Processes index	58%	828

Appendix 3. Decomposition of variance in quality of care

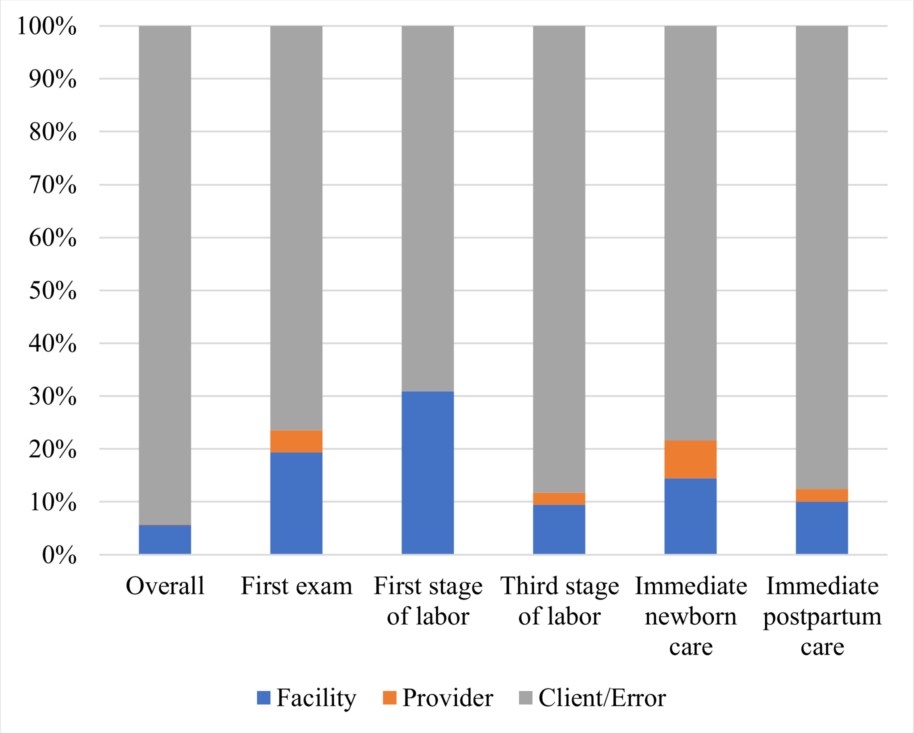

Appendix 4. Quality of care by number of providers

	Quality of care		
Number of providers	Mean	N observations	p-value
1	0.518	371	
2	0.506	317	0.228
3	0.473	109	0.002
4	0.500	27	0.490
5	0.557	4	0.553
Total	0.507	828	

Notes: p-value for difference relative to solo deliveries (1 provider)

Appendix 5. Predictors of having multiple providers during a delivery

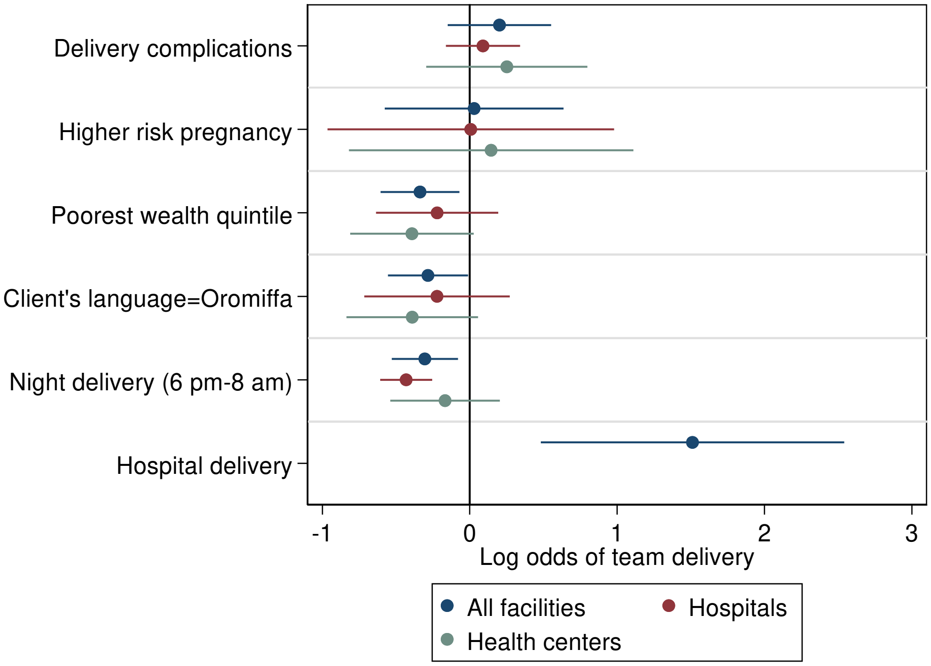

Notes: Points and confidence intervals show the log odds of predictors association with having more than one provider attend the delivery, with observations at the delivery level. Delivery complication is neonatal resuscitation, newborn referred to the NICU, or mother treated for post-partum hemorrhage or eclampsia. Higher risk pregnancy is mother grand multiparous (5 or more births), younger than 18 or older than 35, or has multiple births.

Appendix 6. Bivariate relationships between independent variables and quality of care

	Coef.	p value	95% CI	N
Coworkers' performance across all deliveries	0.059	0.00	(0.04,0.07)	2189
Index provider is most senior in group	-0.056	0.01	(-0.1,-0.02)	2189
Index provider is most experienced in group	0.003	0.82	(-0.02,0.03)	2189
Number of providers	-0.006	0.53	(-0.03,0.01)	2189
Years of experience	0.003	0.44	(0,0.01)	2101
Delivery complication	-0.002	0.92	(-0.04,0.04)	2189
Higher risk pregnancy	-0.001	0.97	(-0.04,0.04)	2186
Night delivery	0.012	0.40	(-0.02,0.04)	2189
Provider cadre (Midwife or nurse diploma ref.)				
Midwife or nurse bsc.	0.047	0.09	(-0.01,0.1)	2149
Health officer	-0.052	0.17	(-0.13,0.02)	2149
GP	-0.037	0.14	(-0.09,0.01)	2149
IESO	0.024	0.85	(-0.22,0.27)	2149
Client wealth index (poorest reference)				
Wealth 2	0.043	0.05	(0,0.09)	1990
Wealth 3	0.077	0.00	(0.03,0.12)	1990
Wealth 4	0.026	0.18	(-0.01,0.06)	1990
Wealth 5 (wealthiest)	0.039	0.05	(0,0.08)	1990
Delivery stage (first exam ref.)				
First stage of labor	-0.001	0.96	(-0.04,0.03)	2189
Third stage of labor	0.228	0.00	(0.2,0.26)	2189
Immediate newborn care	0.294	0.00	(0.25,0.33)	2189
Immediate maternal postpartum care	-0.323	0.00	(-0.36,-0.29)	2189
Facility (Dil Chorra Hospital ref.)				
Sabien Primary Hospital	0.028	0.06	(0,0.06)	2189
Biyowale Health Center	0.067	0.20	(-0.04,0.17)	2189
Legeharae Health Center	0.189	0.00	(0.15,0.23)	2189
Melka Jebdu Health Center	0.139	0.00	(0.09,0.19)	2189
Wahil Health Center	-0.020	0.10	(-0.04,0)	2189
Gende Gerada Health Center	0.034	0.31	(-0.03,0.1)	2189
Goro Health Center	0.197	0.00	(0.16,0.23)	2189
Jelobelina Health Center	0.189	0.00	(0.12,0.26)	2189

Appendix 7. Index provider and coworker provider performance among 2 provider deliveries (N=1326)

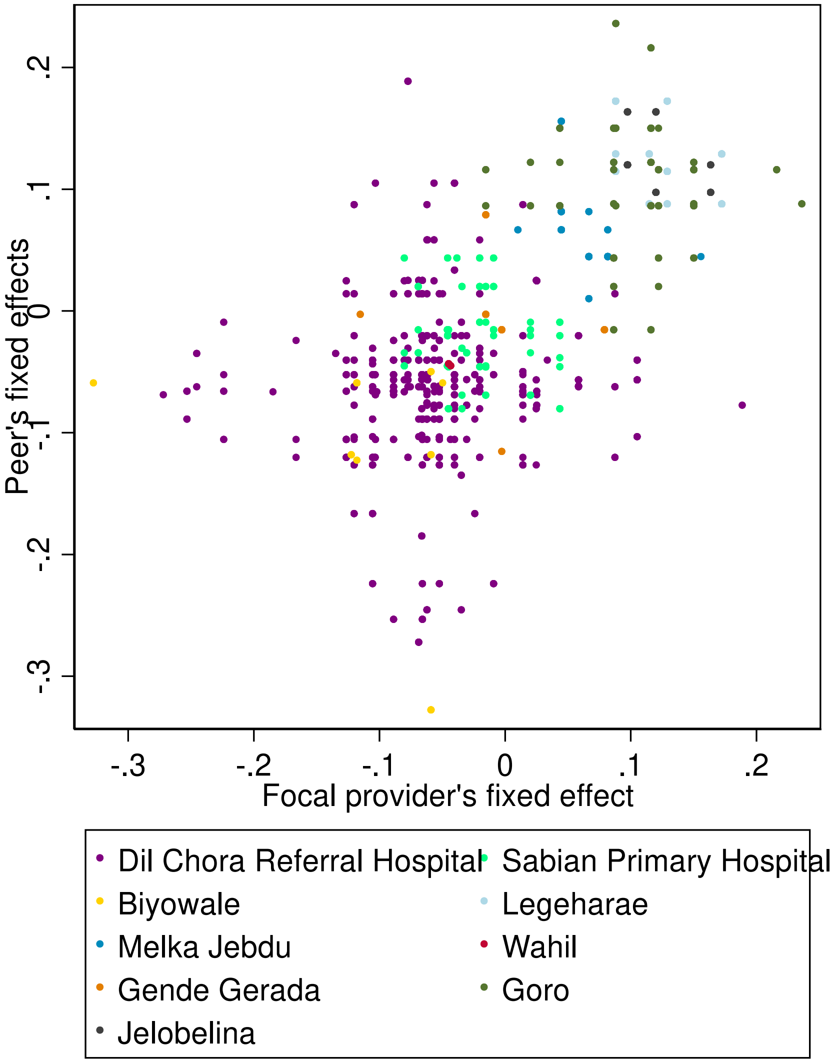

Index provider and coworker provider performance among deliveries with >2 providers (N=674)

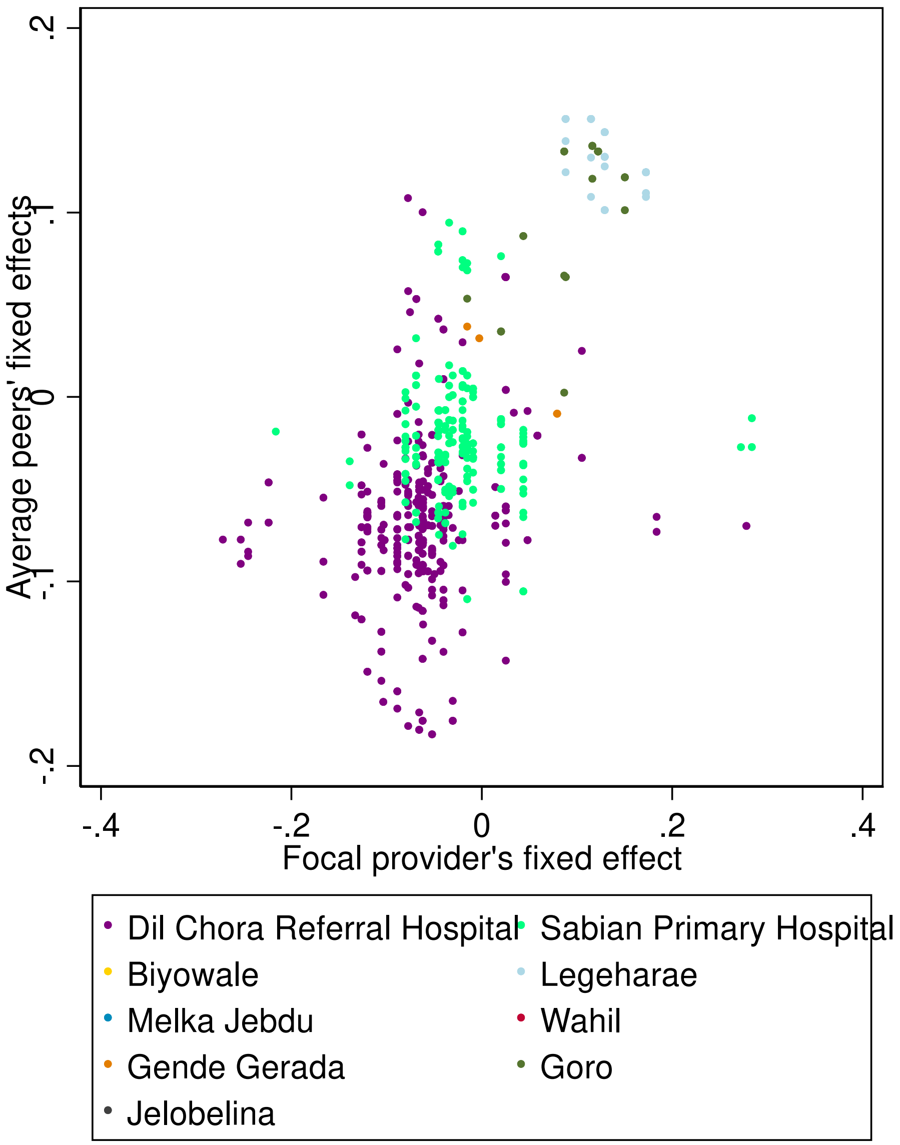
.2
.1
0
-.1
-.2
Average peers' fixed effects
-.4
-.2
0
.2
.4
Focal provider's fixed effect
Dil Chora Referral Hospital
Sabian Primary Hospital
Biyowale
Legeharae
Melka Jebdu
Wahil
Gende Gerada
Goro
Jelobelina

Appendix 8. Multivariable associations between group characteristics and quality of care by delivery stage

	First exam		First stage of labor		Third stage of labor		Immediate newborn		Immediate maternal postpartum	
	Coef.	p value	Coef.	p value	Coef.	p value	Coef.	p value	Coef.	p value
Group characteristics										
Peers' performance across all deliveries	-0.005	0.77	0.042	0.01	0.020	0.10	0.036	0.09	0.023	0.15
Peers are more senior cadre than index	-0.016	0.65	-0.097	0.00	-0.041	0.13	-0.059	0.21	0.004	0.90
Peers are more experienced than index	0.000	0.98	-0.002	0.96	-0.011	0.56	0.000	0.99	-0.030	0.12
Number of providers	-0.011	0.42	0.039	0.05	-0.002	0.89	-0.021	0.34	-0.007	0.61
Index provider characteristics										
Years of experience	-0.001	0.75	0.000	0.92	0.000	0.90	0.000	0.98	-0.003	0.35
Provider cadre (Midwife or nurse diploma ref.)										
Midwife or nurse Bsc.	-0.065	0.05	-0.140	0.00	-0.034	0.20	-0.073	0.16	0.065	0.03
Health officer	-0.142	0.08	-0.016	0.82	-0.161	0.12	-0.506	0.06	0.043	0.72
GP	-0.002	0.97	-0.135	0.00	-0.053	0.13	-0.176	0.00	-0.025	0.52
IESO	-0.206	0.05	-0.088	0.57	0.035	0.77	-0.273	0.09	-0.071	0.09
Context and environment										
Delivery complication	0.044	0.02	-0.039	0.24	0.012	0.66	0.030	0.34	-0.005	0.87
Higher risk pregnancy	-0.022	0.52	0.038	0.28	0.013	0.65	0.003	0.96	-0.006	0.86
Night delivery (morning reference)	0.006	0.75	0.002	0.93	-0.021	0.22	0.021	0.36	0.027	0.22
Client wealth index (poorest reference)										
Wealth 2	0.013	0.67	0.095	0.02	-0.004	0.88	0.040	0.31	0.015	0.60
Wealth 3	0.038	0.22	0.123	0.00	0.066	0.05	0.110	0.02	0.035	0.30
Wealth 4	0.014	0.63	0.066	0.11	0.030	0.37	0.039	0.37	0.043	0.20
Wealth 5 (wealthiest)	0.002	0.94	0.060	0.11	0.046	0.10	0.084	0.06	0.053	0.06
Facility (Dil Chorra Hospital ref.)										
Sabien Primary Hospital	0.156	0.00	0.013	0.62	-0.022	0.51	-0.041	0.42	-0.034	0.29

Biyowale Health Center	-0.150	0.00	0.122	0.36	0.132	0.01	0.335	0.00	0.171	0.23
Legeharae Health Center	0.114	0.01	0.188	0.00	0.116	0.00	0.129	0.06	0.122	0.01
Melka Jebdu Health Center	0.104	0.08	0.173	0.02	0.106	0.00	0.089	0.11	0.101	0.15
Wahil Health Center	0.078	0.09	-0.157	0.00	-0.002	0.94	-0.272	0.00	0.109	0.00
Gende Gerada Health Center	0.085	0.16	0.122	0.00	0.076	0.24	0.217	0.23	0.004	0.97
Goro Health Center	0.152	0.00	0.344	0.00	0.050	0.15	0.178	0.01	-0.003	0.96
Jelobelina Health Center	0.133	0.07	0.243	0.00	0.158	0.01	0.160	0.05	0.106	0.04
Constant	0.470	0.00	0.380	0.00	0.729	0.00	0.778	0.00	0.063	0.34
N obs	454		440		453		387		455	

Appendix 9. Comparison of average vs best coworker performance for deliveries with 3 or more providers

	Coworkers' average performance		Best coworker's performance	
	Coef.	p value	Coef.	p value
Group characteristics				
Coworkers' average performance across all deliveries	0.045	0.01		
Best coworker's performance across all deliveries			0.023	0.08
Coworkers are more senior cadre than index	-0.054	0.04	-0.062	0.02
Coworkers are more experienced than index	-0.024	0.27	-0.023	0.29
Number of providers	0.008	0.68	0.003	0.88
Index provider characteristics				
Years of experience	-0.005	0.25	-0.004	0.29
Provider cadre (Midwife or nurse diploma ref.)				
Midwife or nurse Bsc.	-0.053	0.05	-0.06	0.04
Health officer	-0.173	0.34	-0.181	0.33
GP	-0.088	0.02	-0.093	0.02
IESO	0.001	0.99	-0.004	0.97
Context and environment				
Delivery complication	0.015	0.52	0.017	0.47
Higher risk pregnancy	0.018	0.57	0.018	0.58
Night delivery (morning reference)	0.052	0.01	0.052	0.01
Client wealth index (poorest reference)				
Wealth 2	-0.028	0.40	-0.03	0.37
Wealth 3	0.022	0.47	0.017	0.56
Wealth 4	-0.019	0.57	-0.023	0.50
Wealth 5 (wealthiest)	-0.015	0.63	-0.019	0.55
Delivery stage (first exam ref.)				
First stage of labor	-0.01	0.71	-0.011	0.68
Third stage of labor	0.208	0.00	0.208	0.00
Immediate newborn care	0.247	0.00	0.246	0.00
Immediate postpartum care	-0.335	0.00	-0.335	0.00
Facility (Dil Chorra Hospital ref.)				
Sabien Primary Hospital	0.034	0.23	0.044	0.12
Legeharae Health Center	0.052	0.45	0.102	0.11
Gende Gerada Health Center	0.139	0.16	0.162	0.10
Goro Health Center	0.1	0.09	0.141	0.01
Constant	0.453	0.00	0.46	0.00
N	615		615	

Appendix 10. Association between coworker performance and quality by provider cadre rank

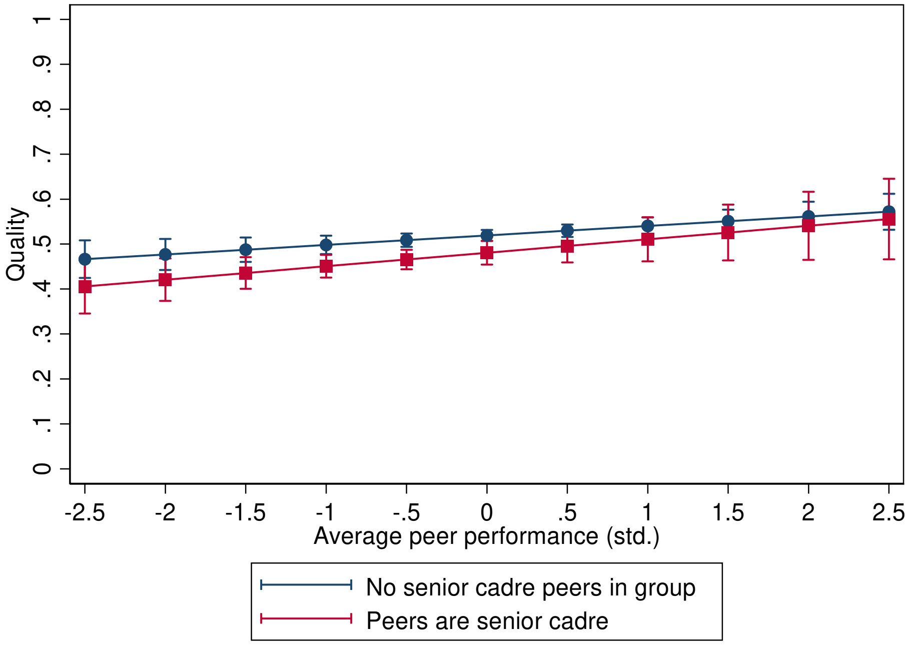

Appendix 11. Association between coworker performance and quality by provider seniority (years of experience)

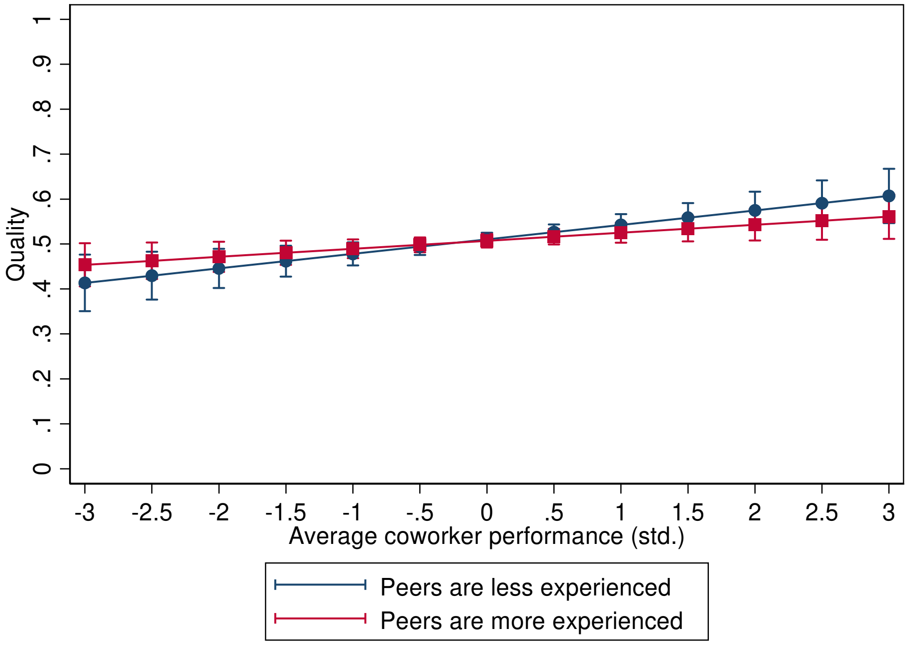

Supplement: Supplementary data [file bmjopen-2022-066111supp001.pdf]
