# Supplementary material for: The role of teams in shaping quality of obstetrical care: a cross-sectional study in Dire Dawa, Ethiopia
Source: BMJ Open. 2022 Oct 3;12(10):e066111. doi: 10.1136/bmjopen-2022-066111 (PMC9535209; doi:10.1136/bmjopen-2022-066111)
Supplement: Author's manuscript [file bmjopen-2022-066111.draft_revisions.pdf]

BMJ Open is committed to open peer review. As part of this commitment we make the peer review history of every article we publish publicly available.

When an article is published we post the peer reviewers' comments and the authors' responses online. We also post the versions of the paper that were used during peer review. These are the versions that the peer review comments apply to.

The versions of the paper that follow are the versions that were submitted during the peer review process. They are not the versions of record or the final published versions. They should not be cited or distributed as the published version of this manuscript.

BMJ Open is an open access journal and the full, final, typeset and author-corrected version of record of the manuscript is available on our site with no access controls, subscription charges or pay-per-view fees (http://bmjopen.bmj.com).

If you have any questions on BMJ Open's open peer review process please email
info.bmjopen@bmj.com

BMJ Open

# The role of teams in shaping quality of obstetric care: a cross-sectional study in Dire Dawa, Ethiopia

| | |
|---|---|
| Journal: | *BMJ Open* |
| Manuscript ID | bmjopen-2022-066111 |
| Article Type: | Original research |
| Date Submitted by the Author: | 01-Jul-2022 |
| Complete List of Authors: | Gage, Anna; Harvard University T H Chan School of Public Health, Global Health and Population; Institute for Health Metrics and Evaluation<br>Yakob, Bereket; The University of British Columbia School of Population and Public Health, Fenot Project; Wolaita Sodo University College<br>McConnell, Margaret; Harvard University T H Chan School of Public Health, Global Health and Population<br>Girma, Tsinuel; The University of British Columbia School of Population and Public Health, Fenot Project<br>Damtachew, Brook; Dil Chora Hospital, Department of Obstetrics and Gynecology<br>Bauhoff, Sebastian; Harvard University T H Chan School of Public Health; Inter-American Development Bank<br>Kruk, Margaret; Harvard University T H Chan School of Public Health, Global Health and Population |
| Keywords: | OBSTETRICS, Public health < INFECTIOUS DISEASES, Human resource management < HEALTH SERVICES ADMINISTRATION & MANAGEMENT, Quality in health care < HEALTH SERVICES ADMINISTRATION & MANAGEMENT |
| | |

SCHOLARONE™
Manuscripts

*I, the Submitting Author has the right to grant and does grant on behalf of all authors of the Work (as defined in the below author licence), an exclusive licence and/or a non-exclusive licence for contributions from authors who are: i) UK Crown employees; ii) where BMJ has agreed a CC-BY licence shall apply, and/or iii) in accordance with the terms applicable for US Federal Government officers or employees acting as part of their official duties; on a worldwide, perpetual, irrevocable, royalty-free basis to BMJ Publishing Group Ltd ("BMJ") its licensees and where the relevant Journal is co-owned by BMJ to the co-owners of the Journal, to publish the Work in this journal and any other BMJ products and to exploit all rights, as set out in our licence.*

*The Submitting Author accepts and understands that any supply made under these terms is made by BMJ to the Submitting Author unless you are acting as an employee on behalf of your employer or a postgraduate student of an affiliated institution which is paying any applicable article publishing charge ("APC") for Open Access articles. Where the Submitting Author wishes to make the Work available on an Open Access basis (and intends to pay the relevant APC), the terms of reuse of such Open Access shall be governed by a Creative Commons licence – details of these licences and which Creative Commons licence will apply to this Work are set out in our licence referred to above.*

*Other than as permitted in any relevant BMJ Author's Self Archiving Policies, I confirm this Work has not been accepted for publication elsewhere, is not being considered for publication elsewhere and does not duplicate material already published. I confirm all authors consent to publication of this Work and authorise the granting of this licence.*

For peer review only

# The role of teams in shaping quality of obstetric care: a cross-sectional study in Dire Dawa, Ethiopia

Anna Gage[1], Bereket Yakob[2,3], Margaret McConnell[1], Tsinuel Girma Nigatu[2], Brook Damtachew[4], Sebastian Bauhoff[1,5], Margaret Kruk[1]

[1]Harvard T.H. Chan School of Public Health, Department of Global Health and Population, Boston, United States
[2]University of British Columbia School of Population and Public Health, Fenot Project, Addis Ababa, Ethiopia
3Woliata Sodo University, School of Public Health, College of Health Sciences and Medicine, Sodo, Ethiopia
[4]Dil Chora Hospital, Department of Obstetrics and Gynecology, Dire Dawa, Ethiopia
[5]Inter-American Development Bank

Corresponding author:
Anna Gage
665 Huntington Ave, Building 1, 11th Floor
Boston, MA 02115
United States
agage@hsph.harvard.edu

For peer review only

## Abstract

Objectives: To examine how characteristics of clinical colleagues influence quality of care.

Design: We conducted a cross-sectional observational study examining the associations between quality of care and provider coworkers, controlling for individual provider's characteristics and contextual factors.

Setting: Nine health facilities in Dire Dawa Administration, Ethiopia from December 2020-February 2021.

Participants: 824 clients and 95 unique providers were observed across the nine health facilities.

Outcome measures: Five phases of the delivery were identified (first exam, first stage of labor, third stage of labor, immediate newborn care, and immediate maternal postpartum care), and a provider was assigned responsibility for the quality of routine care provided during each stage. Defining a provider's coworkers as the other providers who cared for the same client during a different stage, we examined the typical performance of the index provider's coworkers, the seniority of the coworkers' cadre relative to the index provider and the years of experience of the coworkers relative to the index provider.

Results: For the average client, 50% of the recommended routine clinical actions were completed during the delivery overall, with immediate maternal postpartum care being the least well performed (17% of recommended actions). Multiple healthcare providers were involved in 55%



For peer review only - http://bmjopen.bmj.com/site/about/guidelines.xhtml

of deliveries. The number of providers was unassociated with the quality of care but a one standard deviation increase in the coworker's performance was associated with a two-percentage point increase in quality of care ($p<0.01$); this association was largest among providers in the middle quartiles of performance.

Conclusions: A provider's typical performance had a modest positive association with quality of delivery care given by their coworker. As delivery care is often provided by multiple healthcare providers, examining the dynamics of how they influence one another can provide important insights for quality improvement.

**Strengths and limitations of this study**

- This is among the first studies to examine how multiple providers influence one another to provide quality of care in a low-income healthcare setting.
- We use detailed observations of quality of care with actions attributed to specific providers.
- The study examines cross-sectional associations and the effects cannot be interpreted causally.
- We examine quality of routine deliveries only; further research should examine the role of provider groups in managing complications.

For peer review only

## INTRODUCTION

Ethiopia's health system, like those in other low-resource settings, has gaps in maternal and newborn care quality that pose a barrier to improved health outcomes. Although the facility delivery rate has increased dramatically in the past 10 years from 10% in 2011 to 48% in 2019, maternal and newborn mortalities remain high, with 401 maternal deaths per 100,000 live births and 33 neonatal deaths per 1000 live births.[1,2] The majority of such deaths could be avoided with high quality neonatal and maternal care.[3,4] However, in 2018, just 20% of mothers who delivered in a health facility were estimated to receive a high quality of routine maternal care in the Tigray region in northern Ethiopia.[5]

Examinations of poor maternal care quality in similar contexts have found that there is often wide variation in the quality that a single provider will provide across different deliveries. For example, in Uganda the quality of routine actions during labor and delivery varied as much as sixty percentage points across the deliveries for which a single healthcare provider was responsible.[6] In Kenya and Malawi, two studies found that the provider contributed very little to the explained variance in the technical and respectful quality of maternity care.[7,8] The importance of the provider to quality may vary over the course of a delivery, however: another study found that healthcare providers were more important in accounting for the quality of the intrapartum period rather than the assessment or postpartum phases.[9] While these studies have also found that facility, region, and country-level factors also play important roles in determining the quality of care, it is worth further considering why provider quality varies in order to shed light on potential improvement strategies.

One limitation of the above studies in their examination of quality is the focus on attribution of quality to a single healthcare provider over the course of a labor and delivery. While many mothers in Ethiopia receive care from a single healthcare provider, this is not the only model. Rather, multiple healthcare providers may attend a single delivery over the course of the labor, delivery and immediate postpartum period, particularly during more complex cases that lead to higher morbidity and mortality. When groups of providers attend a single patient, their interactions with one another may affect the quality of care.[10] Indeed, the disciplines of complexity science and team science suggest that provider groups are more than the collection of independently acting individuals, but rather they may influence one another in nonlinear and dynamic ways.[10,11] The culmination of these interactions between providers creates the informal group norms and culture around the quality of care that may affect realized quality and health outcomes.

Groups or teams of providers have been examined frequently in healthcare contexts in high-income countries. For example, studies have found that the quality of HIV care is influenced by the performance of a provider's peers on quality[12] and that a provider's patterns of prescribing medications are affected by a specialist in a provider's network.[13] Similarly, studies have found moderate effects of group characteristics such as professional composition and aspects of teamwork such as coordination on quality of care.[14–16] However, these dynamics have not been examined in healthcare in low- and middle-income countries. Using cross-sectional observations of delivery care in Ethiopia and adopting a complex systems lens, this study seeks to understand


For peer review only - http://bmjopen.bmj.com/site/about/guidelines.xhtml

how the number of providers and group dynamics are associated with the quality of delivery care.

## METHODS

### Conceptual framework

We draw on the theory of small groups as complex systems to inform the conceptual framework for this analysis.[11] In this analysis, we define a group of healthcare providers to be all providers who care for a specific woman and newborn over the course of the delivery. Their group identity is thus focused on the shared objective of providing high quality care, and they draw on one another as well as the broader facility environment in achieving this objective.

Arrow et al argue that group behavior involves interactions at three levels.[11] First, behavior is influenced by the constituent elements of the groups, in this case the individual providers who bring different experiences, training, and roles to the group. Second, there are interactions of the group as an entity, which will include feedback loops between group members. Third, there are interactions with the context in which the group is embedded; this context includes the characteristics of the facility as well as the characteristics of the specific delivery for which the group is providing care.

We focused our analysis on the interactions of the group as an entity, with an emphasis on potential spillovers on quality of care. We also account for care context. Drawing on the peer effects literature, we hypothesize several mechanisms by which group members may influence one another in their provision of quality care. First, members may influence one another through hierarchal relationships defined between providers by their cadre rank or years of experience. For example, a provider working with their superior may perform better due to their better supervision or social pressure. Second, there may be informal influence between providers of different abilities. When working with a high performing group member, there may be positive spillovers because of social pressure, knowledge spillovers or social comparison.[17] Conversely, there may negative spillovers due to free riding.[17] While our data does not permit investigation into these specific mechanisms, we assessed whether working with a high performing colleague is associated with higher or lower performance.

### Setting

Dire Dawa is a city administration in Eastern Ethiopia with a population in 2019 of approximately 493,000 people, with 63% living in urban Dire Dawa City and the remaining in rural areas surrounding the City.[18] In the five years preceding 2019, 84% of pregnant women received at least one antenatal care visit and 69% delivered in a health facility.[18] Despite rising utilization rates, neonatal mortality increased between 2011 and 2016 to 36 deaths per 1000 live births.[1] In 2021, the administration had 53 public health facilities: 2 hospitals, 15 health centers and 35 health posts. In addition, there were 5 private hospitals and 7 private clinics.

### Data


For peer review only - http://bmjopen.bmj.com/site/about/guidelines.xhtml

This analysis is part of a broader study to understand the quality of maternal and immediate postpartum care in Dire Dawa Administration's public health system. Cross-sectional primary data was collected in 9 facilities. Public facilities with the highest volumes of deliveries using the 2019 health information system data were included in the study; these facilities were collectively responsible for 83% of the facility deliveries in the region in 2019. The selected facilities consisted of two public hospitals, four health centers in Dire Dawa City and three rural health centers.

Data tools relevant to this analysis include observations of deliveries, interviews with observed clients upon discharge from the facility, and interviews with all providers who provided care. First, all providers who provided intrapartum or immediate postpartum care in the study facilities were invited to take part in a provider survey that asked about their training, perceptions of the working environment and quality of care, and knowledge of complications diagnoses and management.

Second, quality of care was assessed through observations of deliveries by trained health workers. All clients presenting for delivery during the observation period were invited to participate in the study and their care was observed from the time of arrival at the facility until 6 hours postpartum or discharge from the facility. Data collectors identified which items providers completed on a checklist adapted from the Maternal and Child Health Integrated Program tool from USAID. For groups of actions (i.e. first exam), the collector indicated which provider conducted the actions. For this analysis, only deliveries that were observed from admission to discharge were included, so Cesarean section deliveries and intrapartum referrals were excluded. The observation checklist was organized into discrete modules; the ones relevant to this analysis are those for the first exam, checks on the client during the first stage of labor, third stage of labor, immediate newborn care and immediate maternal postpartum care. Finally, all participants whose care was observed were invited to participate in an exit interview upon their discharge from the facility.

Observations of care were conducted in most facilities from December 22, 2020 to February 20, 2021. However, until January 31, 2021, one of the hospitals (Sabian Primary Hospital) was not accepting maternity patients because it was a designated Covid-19 treatment facility. Observations of delivery care in Sabian, therefore, occurred between February 13 and March 21, 2021. Ethical approval for this study was obtained from Harvard University IRB (IRB19-0926), Haramaya University IRB (IHRERC/138/219) and the Ethiopia National Research Ethics and Review Committee (MoSHE//RD/1411/9403/RO).

**Outcome**

The primary outcome of interest for this analysis was the quality of routine maternal care, adapted from the quality of processes of intrapartum and immediate postpartum care index (QoPIIPC).[19] The original index contains 20 indicators of routine actions that should be conducted during every delivery. To attribute the performance of the indicators to a particular provider, we separated this index into five sub-indices associated with the module of the checklist (first exam, first stage of labor, third stage of labor, immediate newborn care and immediate maternal postpartum care). For example, the first exam sub-index consists of seven


For peer review only - http://bmjopen.bmj.com/site/about/guidelines.xhtml

items that should be completed during the first exam such as taking pulse and asking whether the client experienced vaginal bleeding. Index items are presented in Table 1. Performance on each sub-index, which we refer to as the quality of a stage, is calculated as the percent of actions that were completed so it ranges from 0 to 100%. We attributed the quality of each stage for every delivery to the provider who was noted as providing care during that stage; see Appendix 1 for an example of this structure.

**Table 1**. Components of quality of intrapartum and immediate postpartum care processes index by delivery stage

| | Percent complete | N |
|---|---|---|
| Average of first exam actions | 47% | 823 |
| Checks woman's HIV status | 68% | 809 |
| Asks whether woman has experienced headaches or blurred vision | 6% | 823 |
| Asks whether woman has experienced vaginal bleeding | 7% | 823 |
| Takes blood pressure during initial client assessment | 73% | 822 |
| Takes pulse during initial client assessment | 67% | 822 |
| Washes hands before initial examination | 13% | 823 |
| Wears gloves before vaginal examination | 99% | 781 |
| Average of first stage of labor actions | 48% | 795 |
| At least once, explains what will happen in labor | 42% | 824 |
| Prepares uterotonic drug to use for AMTSL | 81% | 791 |
| Uses partograph during labor | 53% | 793 |
| Prepares bags and masks for neonatal resuscitation | 14% | 783 |
| Average of third stage of labor actions | 72% | 822 |
| Correctly administers uterotonic | 56% | 820 |
| Assesses completeness of placenta and membranes | 74% | 819 |
| Assesses for perineal and vaginal membranes | 90% | 819 |
| Ties or clamps cord when pulsations stop, or by 2-3 minutes after birth | 54% | 821 |
| Average of immediate newborn care items | 81% | 694 |
| Immediately dries baby with towel | 98% | 694 |
| Places newborn on mother's abdomen skin-to-skin | 64% | 690 |
| Average of immediate maternal postpartum actions | 17% | 824 |
| Takes mother's vital signs 15 minutes after birth | 0.2% | 823 |

For peer review only

| | | |
|---|---|---|
| Palpates uterus 15 minutes after birth | 19% | 824 |
| Assists mother to initiate breastfeeding | 37% | 695 |
| Quality of Intrapartum and Immediate Postpartum Care Processes index | 51% | 828 |

Notes: AMTSL: Active management of the third stage of labor

Several of the indicators included a timeliness component, for example, whether the provider checked the mother's vital signs 15 minutes after birth. To credit providers who completed these actions outside of the designated timeframe, we also constructed an alternate index without any time limits on the actions as a sensitivity analysis.

13% of delivery observations did not include the module on checks during the first stage of labor. We deemed 30% of these missing observations as "valid missing" if labor was induced directly after the first exam or if there was less than 60 minutes between the end of the first exam and delivery, as there may not have been time for the enumerator to complete the module. For the remaining observations, non-performance of the actions under the first stage (e.g. missing actions imputed as 0) was attributed to the provider who conducted the first exam. Among observations where this stage was not missing, the same provider conducted the first exam and the first stage 65% of the time, while 33% of observations had the same provider for the first and third stages of labor.

**Independent variables**

As described above, each stage of a delivery was assigned to a single responsible provider. We defined a group as the providers that cared for a single client at different stages of her delivery. For deliveries with more than one provider, we were interested in how quality for a particular delivery stage completed by the index provider is associated with the characteristics of other providers in the group. While there may be many other providers working at a facility that are unassociated with a delivery, we define these specific provider groups as the index provider's coworkers for the delivery.

We defined three independent variables of interest. First, we examined the cadre of the coworkers relative to the index provider. We created a binary variable for whether the coworkers had a cadre superior to the index provider. There are five categories of cadres, here ranked from low to high: midwife or nurse (diploma), midwife or nurse (Bsc.), health officer, general practitioner, and integrated emergency surgical officer (IESO). Second, we examined the years of professional experience of the coworkers relative to the index provider. Similar to cadre, we created a binary variable for whether coworkers were more experienced than the index provider.

Third, we defined a measure of coworker performance following a two-step approach.[17] In the first step, we created an individual provider performance score for every provider observed, which captures the index provider's own capabilities. We specified a fixed effects model to estimate the provider's ability across all deliveries and delivery stages where they were the responsible provider. We controlled for delivery stage because of the differences in quality between each stage. The provider's fixed effect became a measure of an individual provider's capabilities.



For peer review only - http://bmjopen.bmj.com/site/about/guidelines.xhtml

In the second step, we took the average of the provider capabilities measure for all other providers who cared for the same client besides the index provider. As a sensitivity analysis for deliveries with three or more providers, we also compared the average coworker performance with the best coworker's performance. The coworker performance measure was standardized for the analysis.

**Covariates**

Drawing on the conceptual framework, we defined several covariates for the individual providers and the contextual environment. First, we included the index provider's cadre and number of years of experience. Second, the context environment included characteristics of the client, the delivery, and the facility. These include whether the birth had a complication (neonatal resuscitation initiated, newborn referred to the NICU, or mother treated for post-partum hemorrhage or eclampsia); whether the delivery was at higher risk for a complication (grand multiparity, mother younger than 18 or older than 35, or multiple births); time of delivery (morning 8 am-6 pm, or night 6 pm-8 am); and the client's wealth (defined by quintiles within the analytic sample using exit interview asset index). Finally, we also controlled for delivery stage as defined above and facility fixed effects.

**Analysis**

We first described the quality of care provided to the study sample on the QoPIIPC index and the characteristics of the clients and providers. We examined whether quality differed by the number of providers caring for the client and examined what factors were associated with group-based care.

We fit a linear mixed-effects model to assess the contribution of group dynamics to quality of care, with observations of delivery stages nested within the index provider. Standard errors were clustered at the index provider. Covariates were missing for a small number of providers and clients; we used multiple imputation such that all observed deliveries meeting the criteria could be included in the analysis. This analysis was conducted among all group deliveries. In addition, we conducted sub-analyses separately for each stage of delivery.

We further investigated how the coworker performance measure interacts with the other group characteristics. First, we categorized the index provider's performance into quintiles and interacted it with coworker performance to understand how relative performance may be associated with quality. Second, we also ran interaction models between coworker performance and the two measures of provider rank (cadre and years of experience) respectively. We used these interaction models to predict quality across the range of coworker performance holding all other covariates at their means and graphed these marginal models.

**Patient and public involvement**

Patients were not involved in the design or implementation of this study.


For peer review only - http://bmjopen.bmj.com/site/about/guidelines.xhtml

## RESULTS

Over the data collection period, 983 clients in the nine facilities were invited to participate in the study. 979 clients (99.6%) agreed to participate, and 828 (84%) were observed throughout the whole delivery and thus met the inclusion criteria for this analysis. The observed clients were cared for by 95 unique providers; 84 (88%) of them were interviewed for the study.

Client and provider characteristics are shown in Table 2. 452 (54%) of the observed deliveries took place at one of the two study hospitals, while the remaining deliveries were at the seven health centers. 213 (26%) of clients experienced a complication during the delivery and 87 (11%) had a higher risk pregnancy. 457 (55%) of deliveries had more than one provider attending over the course of their delivery: 72% of hospital deliveries and 35% of health center deliveries. Among the deliveries with more than one provider, the mean number of providers was 2.4. Two thirds of providers were midwives or nurses (BSc.); providers had an average of 6.2 years of cumulative professional experience.

**Table 2**. Delivery and provider characteristics

| | All deliveries | | Hospitals (N=2) | | Health centers (N=7) | |
|---|---|---|---|---|---|---|
| | N | % | N | % | N | % |
| Client and delivery characteristics | | | | | | |
| N deliveries observed | 828 | | 452 | | 376 | |
| Client's primary language | | | | | | |
| Oromiffa | 484 | 63% | 196 | 49% | 288 | 79% |
| Amharic | 178 | 23% | 129 | 32% | 49 | 13% |
| Somali | 81 | 11% | 52 | 13% | 29 | 8% |
| Other | 23 | 3% | 23 | 6% | 0 | 0% |
| Poorest wealth quintile | 150 | 18% | 52 | 12% | 98 | 26% |
| Experienced complication | 213 | 26% | 69 | 15% | 144 | 38% |
| Higher risk pregnancy | 87 | 11% | 45 | 1% | 42 | 11% |
| Time of delivery | | | | | | |
| Day (8 am-6 pm) | 341 | 41% | 186 | 41% | 155 | 41% |
| Night (6 pm-8 am) | 487 | 59% | 266 | 59% | 221 | 59% |
| Provider characteristics | | | | | | |
| N providers interviewed | 84 | | 50 | | 34 | |
| Years of experience (mean/sd) | 6.2 | 4.5 | 5.9 | 3.6 | 6.5 | 5.7 |
| Female | 54 | 64% | 31 | 62% | 23 | 68% |


For peer review only - http://bmjopen.bmj.com/site/about/guidelines.xhtml

| | | | | | | |
|---|---|---|---|---|---|---|
| Cadre | | | | | | |
| Midwife or Nurse (diploma) | 13 | 15% | 7 | 14% | 6 | 18% |
| Midwife or nurse (BSc) | 55 | 65% | 33 | 66% | 22 | 65% |
| Health officer | 3 | 4% | 0 | 0% | 3 | 9% |
| General practitioner | 7 | 8% | 4 | 8% | 3 | 9% |
| IESO | 6 | 7% | 6 | 12% | 0 | 0% |
| Provider group characteristics | | | | | | |
| Deliveries with >1 provider | 457 | 55% | 326 | 72% | 131 | 35% |
| N providers among group deliveries (mean/sd) | 2.38 | 0.63 | 2.48 | 0.70 | 2.12 | 0.35 |
| At least one health officer, GP or IESO among providers in group | 76 | 16% | 65 | 20% | 11 | 8% |
| At least one provider with over median years of experience | 198 | 43% | 119 | 37% | 79 | 60% |

Notes: Delivery complication is neonatal resuscitation, newborn referred to the NICU, or mother treated for post-partum hemorrhage or eclampsia. Higher risk is mother grand multiparous (5 or more births), younger than 18 or older than 35, or has multiple births. IESO: Integrated Emergency Surgical Officers.

The quality of intrapartum and immediate postpartum care processes provided to the clients in this study was poor (Table 1). During the average delivery, only half of the recommended routine actions were done. Actions ranged from 0.2% of mothers whose vitals were checked fifteen minutes after birth to 98% of babies that were dried immediately with a towel. The recommended actions were most often completed for the immediate newborn care stage, while they were least often done during the immediate maternal postpartum care stage. When the index was defined without any time constraints on the actions, average quality of care rose to 58% of recommended actions across the whole delivery (Appendix 2). However, still only 3% of mothers had their vitals checked after delivery. Over 90% of the variance in quality of care was at the client level, rather than the facility or index provider levels (Appendix 3).

Between 1 and 5 providers cared for a single client and baby over the course of the delivery. The number of providers was unassociated with quality of care (Figure 1 and Appendix 4). The primary determinant of having more than one provider care for a client (Appendix 5) was the hospital delivery. Time of delivery, client wealth and language were slightly associated with a group delivery, but neither higher risk clients nor complicated deliveries was associated with more providers. The remaining results are only among deliveries with more than one provider.

In the bivariate associations between group characteristics and quality of delivery stages (Appendix 6), coworkers' performance and seniority by cadre are associated with quality of care. Scatterplots of the index provider and coworker performance (Appendix 7) further show that this is in part due to quality clustering by facility. Table 3 shows the associations of coworker



For peer review only - http://bmjopen.bmj.com/site/about/guidelines.xhtml

characteristics with quality of care adjusted for all covariates. After controlling for the facility fixed effect, index provider and client characteristics, a one standard deviation increase in coworker performance was associated with a 2-percentage point increase in quality of care during a given delivery stage, or 4% relative to the mean performance. This association was larger during the first stage of labor (4.2 percentage point increase) (Appendix 8). Working with coworkers that were superior in rank to the index provider was associated with 3.5 percentage point lower quality, while the coworker's relative experience was unassociated with quality. In deliveries with three or more providers, the average coworker performance had a higher association with quality than the maximum coworker performance (Appendix 9).

**Table 3.** Group dynamics associations with quality of care (Outcome range 0-1)

| | Quality of care | | |
|---|---|---|---|
| | Coef. | p value | 95% CI |
| Group characteristics | | | |
| Peers' performance across all deliveries | 0.023 | 0.00 | (0.01,0.04) |
| Peers are more senior cadre than index | -0.035 | 0.02 | (-0.07,-0.01) |
| Peers are more experienced than index | -0.009 | 0.46 | (-0.03,0.02) |
| Number of providers | 0.000 | 0.96 | (-0.02,0.02) |
| Index provider characteristics | | | |
| Years of experience | -0.002 | 0.41 | (-0.01,0.00) |
| Provider cadre (Midwife or nurse diploma ref.) | | | |
| Midwife or nurse Bsc. | -0.043 | 0.04 | (-0.09,-0.00) |
| Health officer | -0.141 | 0.02 | (-0.25,-0.03) |
| GP | -0.064 | 0.00 | (-0.11,-0.02) |
| IESO | -0.066 | 0.48 | (-0.25,0.12) |
| Context and environment | | | |
| Delivery complication | 0.004 | 0.81 | (-0.03,0.04) |
| Higher risk pregnancy | 0.003 | 0.88 | (-0.04,0.04) |
| Night delivery (morning reference) | 0.007 | 0.51 | (-0.01,0.03) |
| Client wealth index (poorest reference) | | | |
| Wealth 2 | 0.037 | 0.06 | (-0.00,0.07) |
| Wealth 3 | 0.071 | 0.00 | (0.03,0.11) |
| Wealth 4 | 0.039 | 0.05 | (-0.00,0.08) |
| Wealth 5 (wealthiest) | 0.050 | 0.01 | (0.01,0.09) |
| Delivery stage (first exam ref.) | | | |



For peer review only - http://bmjopen.bmj.com/site/about/guidelines.xhtml

| | | | |
|---|---|---|---|
| First stage of labor | 0.001 | 0.96 | (-0.03,0.04) |
| Delivery care | 0.233 | 0.00 | (0.20,0.26) |
| Immediate newborn care | 0.300 | 0.00 | (0.26,0.34) |
| Immediate postpartum care | -0.320 | 0.00 | (-0.35,-0.29) |
| Facility (Dil Chorra Hospital ref.) | | | |
| Sabien Primary Hospital | 0.017 | 0.35 | (-0.02,0.05) |
| Biyowale Health Center | 0.127 | 0.01 | (0.04,0.22) |
| Legeharae Health Center | 0.129 | 0.00 | (0.07,0.19) |
| Melka Jebdu Health Center | 0.117 | 0.00 | (0.06,0.18) |
| Wahil Health Center | -0.049 | 0.09 | (-0.11,0.01) |
| Gende Gerada Health Center | 0.083 | 0.05 | (0.00,0.16) |
| Goro Health Center | 0.148 | 0.00 | (0.09,0.21) |
| Jelobelina Health Center | 0.160 | 0.00 | (0.09,0.23) |
| Constant | 0.438 | 0.00 | (0.35,0.53) |
| N obs | 2189 | | |

Notes: Regressions are at delivery-stage level, with stages nested within providers. Delivery complication is neonatal resuscitation, newborn referred to the NICU, or mother treated for post-partum hemorrhage or eclampsia. Higher risk is mother grand multiparous (5 or more births), younger than 18 or older than 35, or has multiple births

Given that each subindex contains between 3 and 7 items, these coefficients translate to less than one additional action completed. However, the coefficients are large in comparison to the risk characteristics: deliveries that had a complication or were from higher risk pregnancies were not more likely to have these routine actions completed than less risky deliveries. The quality of care that women in the wealthiest quintile received was on average 5 percentage points higher than women in the poorest wealth quintile.

The marginal associations of coworker performance by the index provider's capabilities are shown in Figure 2. There is no association between coworker performance and quality among the top performing providers: they perform consistently well regardless of their coworkers' performance. However, providers in the middle and low quartiles have large improvement when surrounded by better coworkers. For example, the predicted performance of a third quartile provider when their coworkers' performance is one standard deviation above average is 53% in comparison to 48% when their coworkers are 1 standard deviation below average. Graphs of coworker performance by seniority are included in Appendix 10 and 11; there are not substantive differences in the associations by either cadre rank or years of experience relative to the index provider.

## DISCUSSION



For peer review only - http://bmjopen.bmj.com/site/about/guidelines.xhtml

Deliveries are often attended by multiple providers who must work together to provide high quality care for the mother and newborn, particularly in larger health facilities. Adopting a small groups as complex systems framework, this study examined the provider group dynamics and their associations with quality of care in Dire Dawa, Ethiopia. This study had four key findings. First, the observed quality of routine labor and delivery was poor, especially for postpartum maternal care that is vital in timely diagnosis of potentially fatal conditions such as postpartum hemorrhage. Second, women in the poorest wealth quintiles received worse care, even after controlling for facility and provider characteristics. Third, we found that approximately 70% of hospital deliveries and 35% of health center deliveries had more than one provider involved, but more providers were not associated with higher quality of care. Fourth, providers performed better when working with someone who delivered high quality care, though the differences were small.

We hypothesize several mechanisms by which these provider dynamics may be acting, though our study cannot tease these apart directly. First, we found that if a provider is working with a competent colleague, they do not consequently do less for the client. This is consistent with other peer performance literature, which has shown benefits from working with high-performing colleagues.[12,17] The association with coworker performance was strongest in the first stage of labor, a stage which may require more communication and coordination between providers to share information after the first exam and before delivery. Greater interaction and interdependence between providers during the delivery may therefore create stronger associations. This aligns with recent work demonstrating that increasing the duration and intensity of collaboration between providers can reduce patient mortality.[20] Contrary to our hypothesis, quality of care was lower when coworkers were of a superior cadre to the index provider. Given that the lower cadres provide the routine actions more often than their senior counterparts, there may be some reverse accountability in working with a more diligent junior colleague. Other work has also shown that junior cadres in Ethiopia have smaller gaps between their knowledge and practice.[21]

This work can inform quality improvement in several ways. First, interventions should place an emphasis on improving immediate postpartum maternal care, which has the largest identified gaps and the greatest potential for reducing severe maternal morbidity and mortality. While the gaps in monitoring vitals and palpating the uterus that we found in Dire Dawa are larger than those seen in other areas of Ethiopia, the immediate postpartum period has previously been identified as a neglected area.[22,23] Indeed, in evaluations of quality improvement interventions in Ethiopia, the mother's postpartum care is often not even included as an outcome in favor of immediate newborn care.[24,25] Interventions may emphasize this area in health worker education or regular supervision. Second, the propensity for multiple providers during delivery suggests that health worker education should emphasize team-based models of care, perhaps through team drills for emergency scenarios. Knowing how to work as a team, assign roles and responsibilities and communicate findings may improve the performance of the team.[26] While our study assessed mainly normal births, highly optimized team-based care will also be required to reduce mortality in the case of obstetric emergencies.[27]

Third, improvement interventions may consider management or supervision approaches that pair providers of mixed competency levels. Our analysis shows that only the very top performers do


For peer review only - http://bmjopen.bmj.com/site/about/guidelines.xhtml

not benefit from their coworkers' performance, while most providers may benefit from working with a high performer. Arranging staffing schedules to intentionally pair higher and lower providers may increase the potential for these positive spillovers and strengthen accountability. Supervision approaches typically use traditional cadre hierarchies to define who may act as a supervisor;[28] however, this work suggests that junior cadres may be able to effectively supervise more senior coworkers. Alternately, actual performance could be used to identify potential supervisors.

Finally, the culmination of these interactions between providers over many deliveries creates the facility culture and norms for quality of care. Interventions that aim to change facility norms on quality of care, such as group problem solving or quality improvement collaboratives have shown moderate to high effects on health worker practices in meta-analyses.[29,30] For example, an improvement intervention in India that used an integrated training, mentoring and a plan-do-study-act cycle with an emphasis on peer learning improved postpartum monitoring of mothers from 52% to 94%.[31] However, this study also shows the limits of micro-focused interventions. The potential for a two-percentage point improvement from pairing providers will not overcome the enormous quality deficit seen in labor and delivery care in Dire Dawa. Rather, other macro level strategies such as improved pre-service education or redesigning maternal and newborn care service to be provided at CEmONC capable facilities will likely be necessary.[32,33]

This study is among the first to examine how multiple providers influence one another to provide quality of care in a low-income healthcare setting. The detailed observation data with information on who attended each stage of delivery is a strength of the analysis. There are, however, several limitations to address in future research. First, missing provider attribution for the first stage of delivery required us to make assumptions about who was responsible for the care that was not provided. Second, although we controlled for many potential confounders, the associations may not be interpreted causally. The formation of the provider groups may in some cases be endogenous, for example if a proactive provider actively seeks support from a high-quality provider to assist with a complex delivery. Third, this study only focused on routine actions that should be done for every mother and newborn. However, the quality of complications management is likely both more impactful for consequent health outcomes and often requires multiple providers to work together coincidentally rather than sequentially. Future research should examine the coordination and communications of providers working to address complications. Finally, this study took place during the Covid-19 pandemic in Ethiopia and for a period during the study, one of the two public hospitals in Dira Dawa was closed to deliveries because it was serving as a Covid treatment facility. This resulted in unusually high delivery volumes in the other hospital which may have altered provider dynamics.

## CONCLUSION

Over the past decade, approximately 31% of deliveries in Sub-Saharan Africa took place in hospitals and there are calls to increase this proportion.[34,35] As more women deliver in hospitals, they are more likely to be attended by multiple healthcare providers, which could have consequences for the quality of care they receive. Unpacking the provider dynamics in how they work together to deliver care quality can yield useful insights for quality improvement in the future.



For peer review only - http://bmjopen.bmj.com/site/about/guidelines.xhtml

**Authorship statement:** ADG, MEK and BY conceived the study. BY, BD and TNG oversaw the data collection. ADG conducted the analysis and wrote the first draft with substantial input from all authors. All authors critically reviewed the draft and approved this version for submission.

**Competing Interests:** None declared.

**Funding:** We acknowledge funding from the Bill & Melinda Gates Foundation [Grant No. OPP1135922].

**Data sharing statement:** Data available from corresponding author upon reasonable request.

**Ethics:** Ethical approval for this study was obtained from Harvard University IRB, Haramaya University IRB and the Ethiopia National Research Ethics and Review Committee.

**Acknowledgements:** We gratefully acknowledge the contributions from the data collectors and study participants.

For peer review only



For peer review only - http://bmjopen.bmj.com/site/about/guidelines.xhtml

## References


1 Ethiopian Public Health Institute. Ethiopia Mini Demographic and Health Survey. Addis Ababa, 2021 https://dhsprogram.com/pubs/pdf/FR363/FR363.pdf (accessed Nov 4, 2021).

2 World Bank. World Development Indicators. 2017. http://databank.worldbank.org/data/reports.aspx?source=World-Development-Indicators# (accessed Feb 10, 2018).

3 Tessema GA, Laurence CO, Melaku YA, *et al.* Trends and causes of maternal mortality in Ethiopia during 1990-2013: findings from the Global Burden of Diseases study 2013. *BMC public health* 2017; **17**: 160.

4 Berhan Y, Berhan A. Perinatal mortality trends in Ethiopia. *Ethiopian journal of health sciences* 2014; **24**: 29–40.

5 Weldearegay HG, Kahsay AB, Medhanyie AA, Godefay H, Petrucka P. Quality of and barriers to routine childbirth care signal functions in primary level facilities of Tigray, Northern Ethiopia: Mixed method study. *PloS one* 2020; **15**: e0234318.

6 Rokicki S, Mwesigwa B, Cohen JL. Know-do gaps in obstetric and newborn care quality in Uganda: a cross-sectional study in rural health facilities. *Tropical Medicine & International Health* 2021; **26**: 535–45.

7 Arsenault C, English M, Gathara D, Malata A, Mandala W, Kruk ME. Variation in competent and respectful delivery care in Kenya and Malawi: a retrospective analysis of national facility surveys. *Tropical Medicine & International Health* 2020; **25**: 442–53.

8 Helfinstein S, Jain M, Ramesh BM, *et al.* Facilities are substantially more influential than care providers in the quality of delivery care received: a variance decomposition and clustering analysis in Kenya, Malawi and India. *BMJ global health* 2020; **5**: e002437.

9 Owili PO, Muga MA, Mendez BR, Chen B. Quality of maternity care and its determinants along the continuum in Kenya: A structural equation modeling analysis. *PLoS One* 2017; **12**: e0177756.




For peer review only - http://bmjopen.bmj.com/site/about/guidelines.xhtml

10 Mach M, Abrantes AC, Soler C. Teamwork in Healthcare Management. *Teamwork in Healthcare* 2021; : 23.

11 Arrow H, McGrath JE, Berdahl JL. Small groups as complex systems: Formation, coordination, development, and adaptation. Sage Publications, 2000.

12 Stecher C. Physician network connections to specialists and HIV quality of care. *Health Services Research* 2021.

13 Nair HS, Manchanda P, Bhatia T. Asymmetric social interactions in physician prescription behavior: The role of opinion leaders. *Journal of Marketing Research* 2010; **47**: 883–95.

14 Schmutz JB, Meier LL, Manser T. How effective is teamwork really? The relationship between teamwork and performance in healthcare teams: a systematic review and meta-analysis. *BMJ open* 2019; **9**: e028280.

15 Rosen MA, DiazGranados D, Dietz AS, *et al.* Teamwork in healthcare: Key discoveries enabling safer, high-quality care. *American Psychologist* 2018; **73**: 433.

16 Gharaveis A, Hamilton DK, Pati D. The impact of environmental design on teamwork and communication in healthcare facilities: a systematic literature review. *HERD: Health Environments Research & Design Journal* 2018; **11**: 119–37.

17 Tan TF, Netessine S. When you work with a superman, will you also fly? an empirical study of the impact of coworkers on performance. *Management Science* 2019; **65**: 3495–517.

18 UNICEF. Situation Analysis of Children and Women: Dire Dawa Administration. 2019 https://www.unicef.org/ethiopia/media/2556/file/Dire%20Dawa.pdf (accessed Sept 29, 2021).

19 Tripathi V, Stanton C, Strobino D, Bartlett L. Development and Validation of an Index to Measure the Quality of Facility-Based Labor and Delivery Care Processes in Sub-Saharan Africa. *PLoS One* 2015; **10**: e0129491.

20 Chen Y. Team-specific human capital and team performance: Evidence from doctors. *American Economic Review* 2019.

For peer review only



For peer review only - http://bmjopen.bmj.com/site/about/guidelines.xhtml

21 Gage AD, Kruk ME, Girma T, Lemango ET. The know-do gap in sick child care in Ethiopia. *PLoS One* 2018; **13**: e0208898.

22 Kebede AA, Taye BT, Wondie KY, Tiguh AE, Eriku GA, Mihret MS. Healthcare provider's adherence to immediate postpartum care guidelines in Gondar province hospitals, northwest Ethiopia: A multicenter study. *Plos one* 2021; **16**: e0259263.

23 Fisseha G, Berhane Y, Worku A, Terefe W. Quality of the delivery services in health facilities in Northern Ethiopia. *BMC health services research* 2017; **17**: 1–7.

24 Magge H, Kiflie A, Nimako K, *et al.* The Ethiopia healthcare quality initiative: design and initial lessons learned. *International Journal for Quality in Health Care* 2019; **31**: G180–6.

25 Hagaman AK, Singh K, Abate M, *et al.* The impacts of quality improvement on maternal and newborn health: preliminary findings from a health system integrated intervention in four Ethiopian regions. *BMC health services research* 2020; **20**: 1–12.

26 Fransen AF, de Ven J, Banga FR, Mol BWJ, Oei SG. Multi-professional simulation-based team training in obstetric emergencies for improving patient outcomes and trainees' performance. *Cochrane Database of Systematic Reviews* 2020.

27 Dalby P, Gosman GG, Stein K, Streitman D, Wise N. Crisis teams for obstetric patients. *Textbook of Rapid Response Systems* 2017; : 229–39.

28 Austin A, Gulema H, Belizan M, *et al.* Barriers to providing quality emergency obstetric care in Addis Ababa, Ethiopia: healthcare providers' perspectives on training, referrals and supervision, a mixed methods study. *BMC pregnancy and childbirth* 2015; **15**: 1–10.

29 Rowe AK, Rowe SY, Peters DH, Holloway KA, Chalker J, Ross-Degnan D. Effectiveness of strategies to improve health-care provider practices in low-income and middle-income countries: a systematic review. *The Lancet Global Health* 2018; **6**: e1163–75.

30 Garcia-Elorrio E, Rowe SY, Teijeiro ME, Ciapponi A, Rowe AK. The effectiveness of the quality improvement collaborative strategy in low- and middle-income countries: A systematic review and meta-analysis. *PloS one* 2019; **14**: e0221919.



For peer review only - http://bmjopen.bmj.com/site/about/guidelines.xhtml

31 Taneja G, Sarin E, Bajpayee D, *et al.* Care Around Birth Approach: A Training, Mentoring, and Quality Improvement Model to Optimize Intrapartum and Immediate Postpartum Quality of Care in India. *Global Health: Science and Practice* 2021; **9**: 590–610.

32 Nimako K, Kruk ME. Seizing the moment to rethink health systems. *The Lancet Global Health* 2021.

33 Kruk ME, Gage AD, Arsenault C, *et al.* High-quality health systems in the Sustainable Development Goals era: time for a revolution. *The Lancet Global Health* 2018; **6**: e1196–252.

34 Gage AD, Fink G, Ataguba JE, Kruk ME. Hospital delivery and neonatal mortality in 37 countries in sub-Saharan Africa and South Asia: An ecological study. *PLOS Medicine* 2021; **18**: e1003843.

35 Roder-DeWan S, Nimako K, Twum-Danso NA, Amatya A, Langer A, Kruk M. Health system redesign for maternal and newborn survival: rethinking care models to close the global equity gap. *BMJ Global Health* 2020; **5**: e002539.

For peer review only


For peer review only - http://bmjopen.bmj.com/site/about/guidelines.xhtml

**Figure 1**. Quality of intrapartum and immediate postpartum care processes by number of providers and facility type

**Figure 2**. Predicted quality of care by coworker performance and index provider performance
Notes: Points show the predicted estimate of quality by their coworkers' performance for the given level of index provider performance; bars show 95% confidence intervals. Gray line shows median performance.

For peer review only

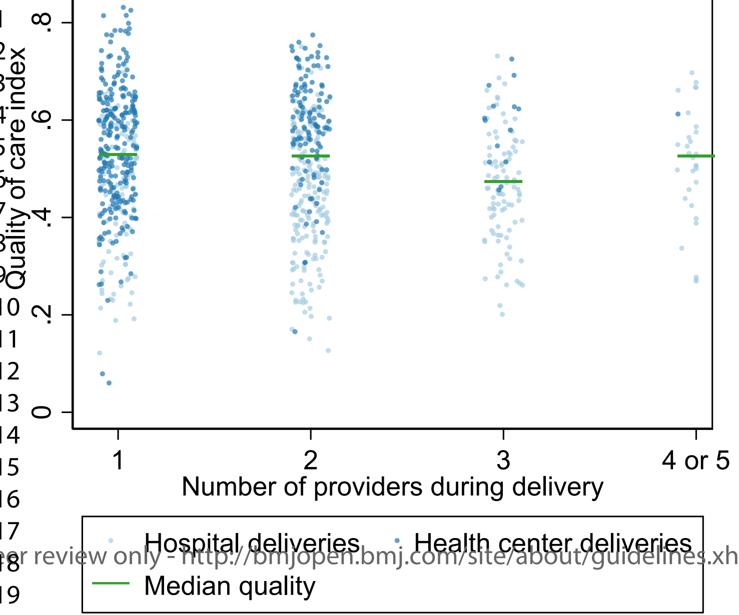


For peer review only - http://bmjopen.bmj.com/site/about/guidelines.xhtml

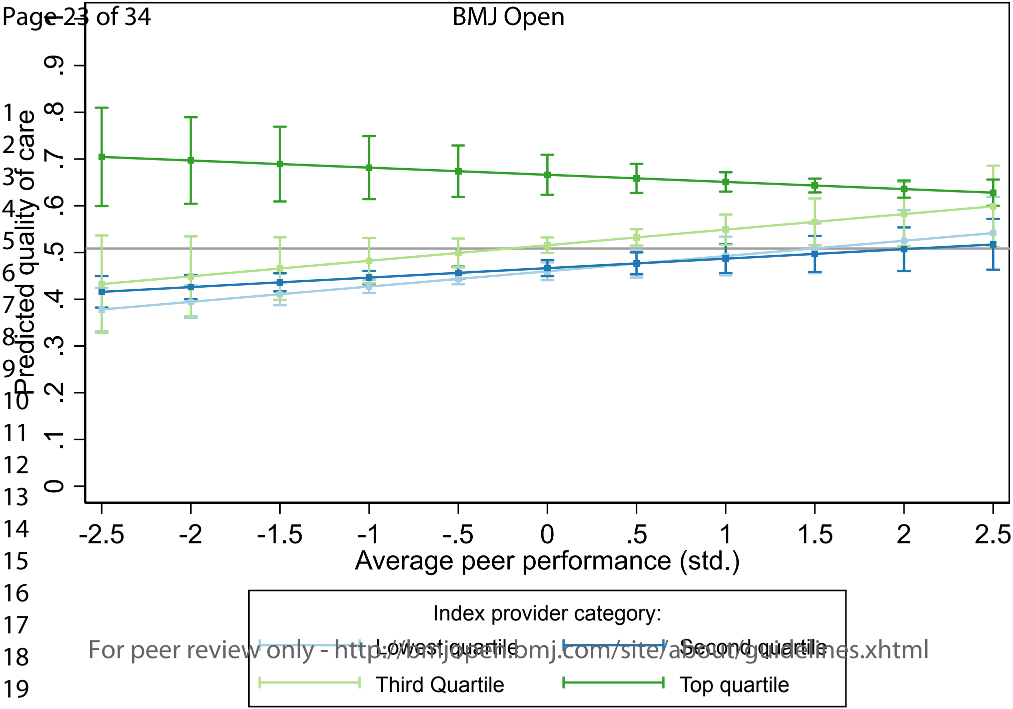


For peer review only - http://bmjopen.bmj.com/site/about/guidelines.xhtml

**Appendix 1.** Examples of provider arrangements during labor and delivery

| Number of providers | First exam | First stage of labor | Delivery care | Immediate newborn care | Immediate maternal postpartum care | Frequency |
|---|---|---|---|---|---|---|
| 1 | | | | | | 45% |
| 2 | | | | | | 38% |
| 3 | | | | | | 13% |
| 4 | | | | | | 3% |
| 5 | | | | | | 0.5% |

| Legend |
|---|
| Provider 1 |
| Provider 2 |
| Provider 3 |
| Provider 4 |
| Provider 5 |

For peer review only

**Appendix 2.** Quality of intrapartum and immediate postpartum care processes with no time constraints on actions

| | Percent completed | N |
|---|---|---|
| Average of first exam actions | 47% | 823 |
| Checks woman's HIV status | 68% | 809 |
| Asks whether woman has experienced headaches or blurred vision | 6% | 823 |
| Asks whether woman has experienced vaginal bleeding | 7% | 823 |
| Takes blood pressure during initial client assessment | 73% | 822 |
| Takes pulse during initial client assessment | 67% | 822 |
| Washes hands before initial examination | 13% | 823 |
| Wears gloves before vaginal examination | 99% | 781 |
| Average of first stage of labor actions | 48% | 795 |
| At least once, explains what will happen in labor | 42% | 824 |
| Prepares uterotonic drug to use for AMTSL | 81% | 791 |
| Uses partograph during labor | 53% | 793 |
| Prepares bags and masks for neonatal resuscitation | 14% | 783 |
| Average of third stage of labor actions | 88% | 828 |
| Correctly administers uterotonic (no time requirement) | 93% | 820 |
| Assesses completeness of placenta and membranes | 74% | 819 |
| Assesses for perineal and vaginal membranes | 90% | 819 |
| Ties or clamps cord | 99% | 828 |
| Average of immediate newborn care | 81% | 694 |
| Immediately dries baby with towel | 98% | 694 |
| Places newborn on mother's abdomen skin-to-skin | 64% | 690 |
| Average of immediate maternal postpartum stage actions | 37% | 824 |
| Takes mother's vital signs after birth | 3% | 823 |
| Palpates uterus after birth | 72% | 824 |
| Assists mother to initiate breastfeeding | 37% | 695 |

| | | |
|---|---|---|
| Alternate Quality of Intrapartum and Immediate Postpartum Care Processes index | 58% | 828 |

**Appendix 3**. Decomposition of variance in quality of care

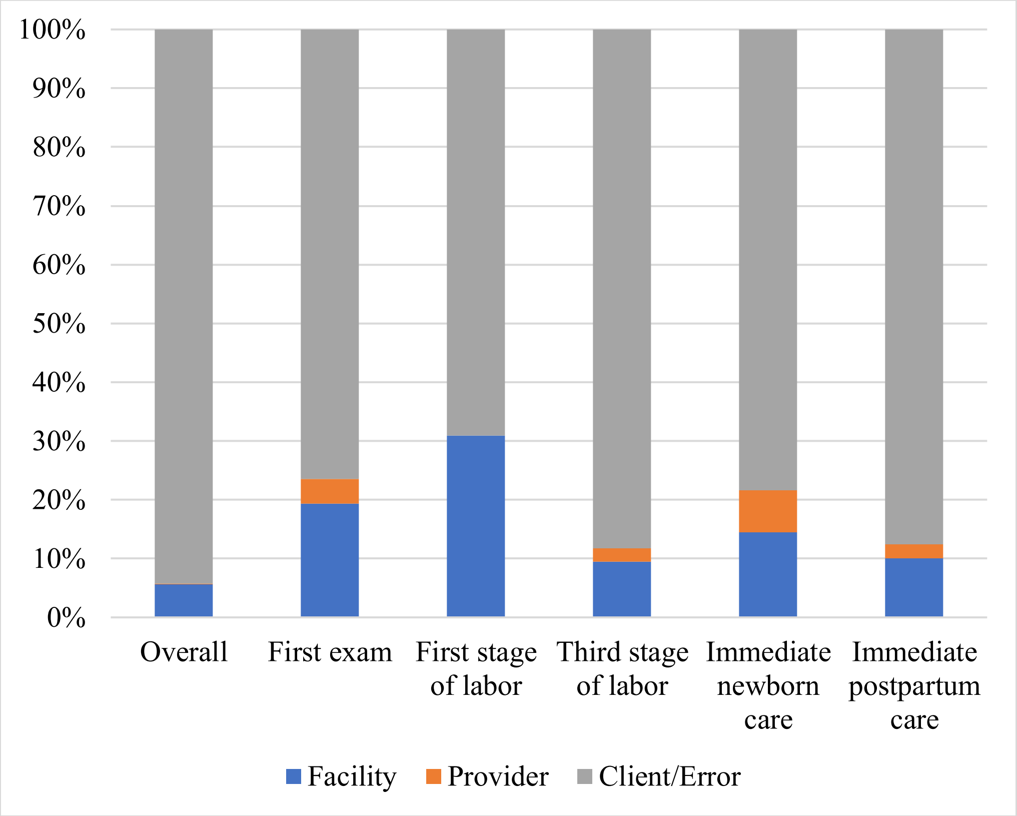


**Appendix 4.** Quality of care by number of providers

| | Quality of care | | |
|---|---|---|---|
| Number of providers | Mean | N observations | p-value |
| 1 | 0.518 | 371 | |
| 2 | 0.506 | 317 | 0.228 |
| 3 | 0.473 | 109 | 0.002 |
| 4 | 0.500 | 27 | 0.490 |
| 5 | 0.557 | 4 | 0.553 |
| Total | 0.507 | 828 | |

Notes: p-value for difference relative to solo deliveries (1 provider)

For peer review only - http://bmjopen.bmj.com/site/about/guidelines.xhtml

**Appendix 5.** Predictors of having multiple providers during a delivery

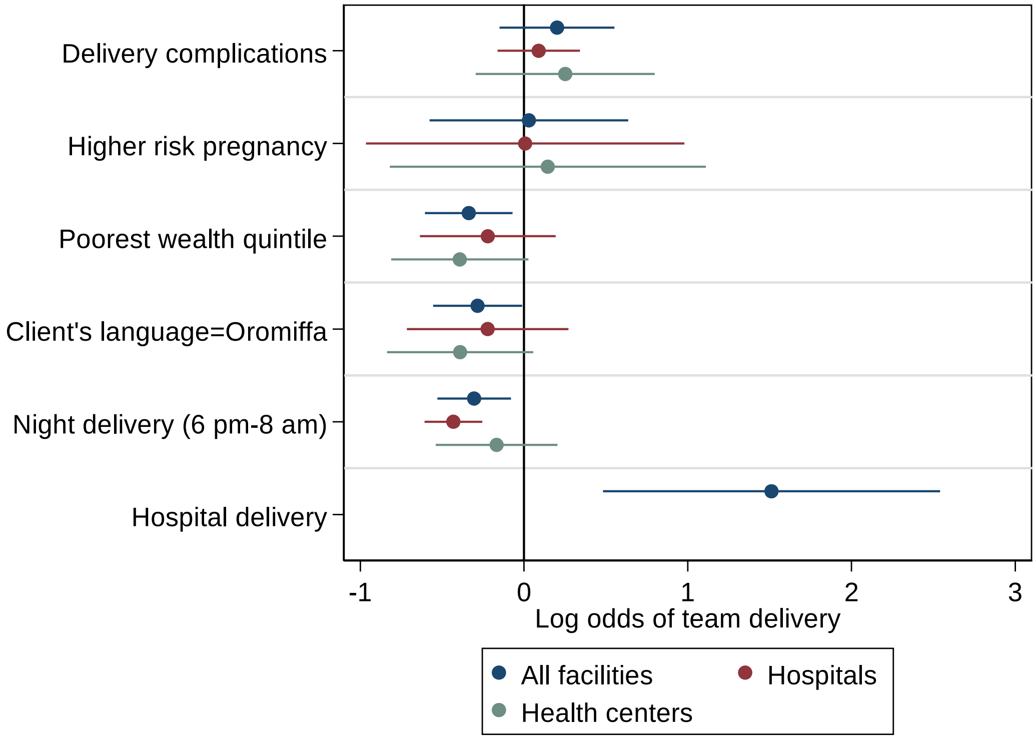


Notes: Points and confidence intervals show the log odds of predictors association with having more than one provider attend the delivery, with observations at the delivery level. Delivery complication is neonatal resuscitation, newborn referred to the NICU, or mother treated for post-partum hemorrhage or eclampsia. Higher risk pregnancy is mother grand multiparous (5 or more births), younger than 18 or older than 35, or has multiple births.

For peer review only

**Appendix 6.** Index provider and coworker provider performance among 2 provider deliveries (N=1326)

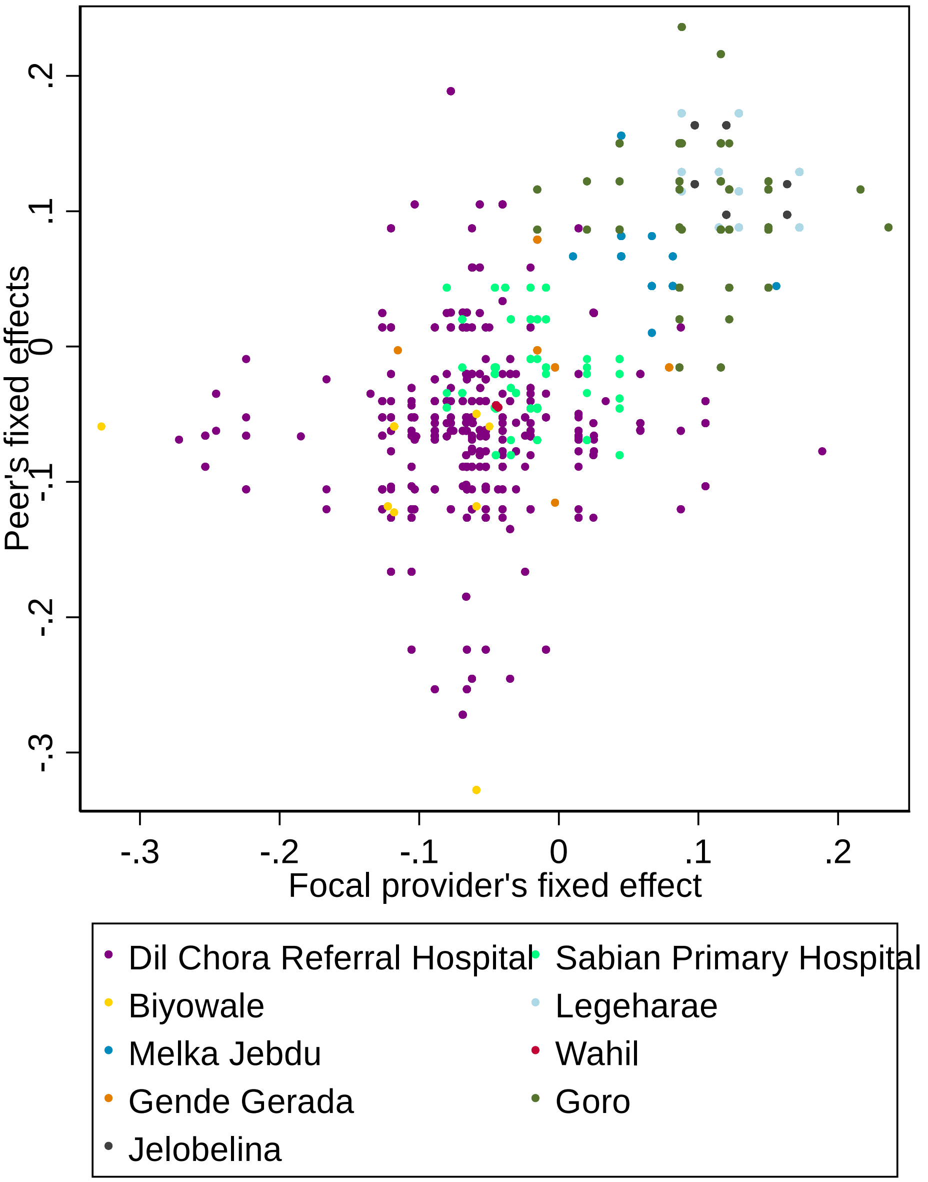


Index provider and coworker provider performance among deliveries with >2 providers (N=674)

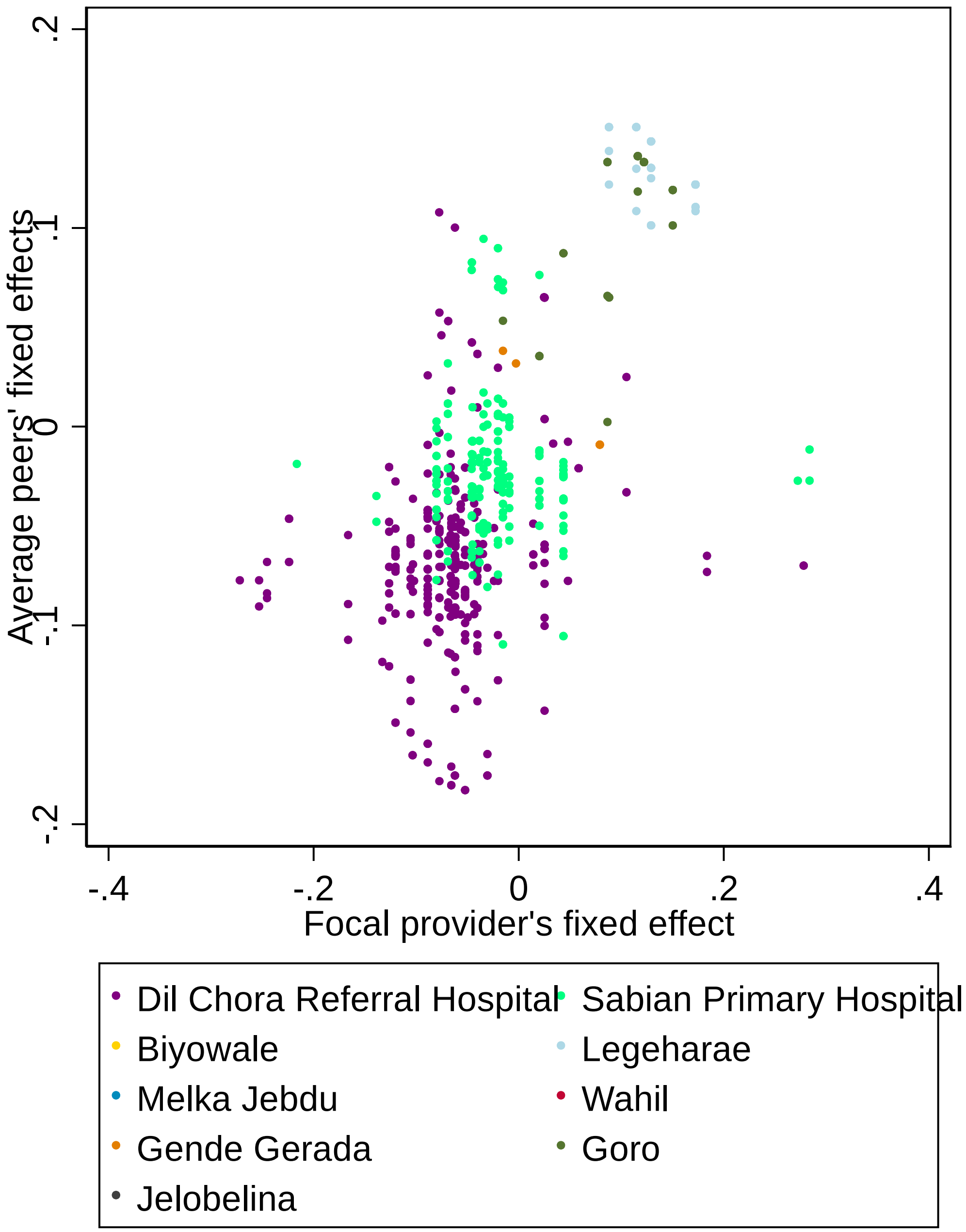
.2
.1
0
-.1
-.2
Average peers' fixed effects
-.4
-.2
0
.2
.4
Focal provider's fixed effect
Dil Chora Referral Hospital
Sabian Primary Hospital
Biyowale
Legeharae
Melka Jebdu
Wahil
Gende Gerada
Goro
Jelobelina

**Appendix 7.** Bivariate relationships between independent variables and quality of care

| | Coef. | p value | 95% CI | N |
|---|---|---|---|---|
| Coworkers' performance across all deliveries | 0.059 | 0.00 | (0.04,0.07) | 2189 |
| Index provider is most senior in group | -0.056 | 0.01 | (-0.1,-0.02) | 2189 |
| Index provider is most experienced in group | 0.003 | 0.82 | (-0.02,0.03) | 2189 |
| Number of providers | -0.006 | 0.53 | (-0.03,0.01) | 2189 |
| Years of experience | 0.003 | 0.44 | (0,0.01) | 2101 |
| Delivery complication | -0.002 | 0.92 | (-0.04,0.04) | 2189 |
| Higher risk pregnancy | -0.001 | 0.97 | (-0.04,0.04) | 2186 |
| Night delivery | 0.012 | 0.40 | (-0.02,0.04) | 2189 |
| Provider cadre (Midwife or nurse diploma ref.) | | | | |
| Midwife or nurse bsc. | 0.047 | 0.09 | (-0.01,0.1) | 2149 |
| Health officer | -0.052 | 0.17 | (-0.13,0.02) | 2149 |
| GP | -0.037 | 0.14 | (-0.09,0.01) | 2149 |
| IESO | 0.024 | 0.85 | (-0.22,0.27) | 2149 |
| Client wealth index (poorest reference) | | | | |
| Wealth 2 | 0.043 | 0.05 | (0,0.09) | 1990 |
| Wealth 3 | 0.077 | 0.00 | (0.03,0.12) | 1990 |
| Wealth 4 | 0.026 | 0.18 | (-0.01,0.06) | 1990 |
| Wealth 5 (wealthiest) | 0.039 | 0.05 | (0,0.08) | 1990 |
| Delivery stage (first exam ref.) | | | | |
| First stage of labor | -0.001 | 0.96 | (-0.04,0.03) | 2189 |
| Third stage of labor | 0.228 | 0.00 | (0.2,0.26) | 2189 |
| Immediate newborn care | 0.294 | 0.00 | (0.25,0.33) | 2189 |
| Immediate maternal postpartum care | -0.323 | 0.00 | (-0.36,-0.29) | 2189 |
| Facility (Dil Chorra Hospital ref.) | | | | |
| Sabien Primary Hospital | 0.028 | 0.06 | (0,0.06) | 2189 |
| Biyowale Health Center | 0.067 | 0.20 | (-0.04,0.17) | 2189 |
| Legeharae Health Center | 0.189 | 0.00 | (0.15,0.23) | 2189 |
| Melka Jebdu Health Center | 0.139 | 0.00 | (0.09,0.19) | 2189 |
| Wahil Health Center | -0.020 | 0.10 | (-0.04,0) | 2189 |
| Gende Gerada Health Center | 0.034 | 0.31 | (-0.03,0.1) | 2189 |
| Goro Health Center | 0.197 | 0.00 | (0.16,0.23) | 2189 |
| Jelobelina Health Center | 0.189 | 0.00 | (0.12,0.26) | 2189 |

For peer review only

**Appendix 8.** Multivariable associations between group characteristics and quality of care by delivery stage

| | First exam | | First stage of labor | | Third stage of labor | | Immediate newborn | | Immediate maternal postpartum | |
|---|---|---|---|---|---|---|---|---|---|---|
| | Coef. | p value | Coef. | p value | Coef. | p value | Coef. | p value | Coef. | p value |
| Group characteristics | | | | | | | | | | |
| Peers' performance across all deliveries | -0.005 | 0.77 | 0.042 | 0.01 | 0.020 | 0.10 | 0.036 | 0.09 | 0.023 | 0.15 |
| Peers are more senior cadre than index | -0.016 | 0.65 | -0.097 | 0.00 | -0.041 | 0.13 | -0.059 | 0.21 | 0.004 | 0.90 |
| Peers are more experienced than index | 0.000 | 0.98 | -0.002 | 0.96 | -0.011 | 0.56 | 0.000 | 0.99 | -0.030 | 0.12 |
| Number of providers | -0.011 | 0.42 | 0.039 | 0.05 | -0.002 | 0.89 | -0.021 | 0.34 | -0.007 | 0.61 |
| Index provider characteristics | | | | | | | | | | |
| Years of experience | -0.001 | 0.75 | 0.000 | 0.92 | 0.000 | 0.90 | 0.000 | 0.98 | -0.003 | 0.35 |
| Provider cadre (Midwife or nurse diploma ref.) | | | | | | | | | | |
| Midwife or nurse Bsc. | -0.065 | 0.05 | -0.140 | 0.00 | -0.034 | 0.20 | -0.073 | 0.16 | 0.065 | 0.03 |
| Health officer | -0.142 | 0.08 | -0.016 | 0.82 | -0.161 | 0.12 | -0.506 | 0.06 | 0.043 | 0.72 |
| GP | -0.002 | 0.97 | -0.135 | 0.00 | -0.053 | 0.13 | -0.176 | 0.00 | -0.025 | 0.52 |
| IESO | -0.206 | 0.05 | -0.088 | 0.57 | 0.035 | 0.77 | -0.273 | 0.09 | -0.071 | 0.09 |
| Context and environment | | | | | | | | | | |
| Delivery complication | 0.044 | 0.02 | -0.039 | 0.24 | 0.012 | 0.66 | 0.030 | 0.34 | -0.005 | 0.87 |
| Higher risk pregnancy | -0.022 | 0.52 | 0.038 | 0.28 | 0.013 | 0.65 | 0.003 | 0.96 | -0.006 | 0.86 |
| Night delivery (morning reference) | 0.006 | 0.75 | 0.002 | 0.93 | -0.021 | 0.22 | 0.021 | 0.36 | 0.027 | 0.22 |
| Client wealth index (poorest reference) | | | | | | | | | | |
| Wealth 2 | 0.013 | 0.67 | 0.095 | 0.02 | -0.004 | 0.88 | 0.040 | 0.31 | 0.015 | 0.60 |
| Wealth 3 | 0.038 | 0.22 | 0.123 | 0.00 | 0.066 | 0.05 | 0.110 | 0.02 | 0.035 | 0.30 |
| Wealth 4 | 0.014 | 0.63 | 0.066 | 0.11 | 0.030 | 0.37 | 0.039 | 0.37 | 0.043 | 0.20 |
| Wealth 5 (wealthiest) | 0.002 | 0.94 | 0.060 | 0.11 | 0.046 | 0.10 | 0.084 | 0.06 | 0.053 | 0.06 |
| Facility (Dil Chorra Hospital ref.) | | | | | | | | | | |
| Sabien Primary Hospital | 0.156 | 0.00 | 0.013 | 0.62 | -0.022 | 0.51 | -0.041 | 0.42 | -0.034 | 0.29 |

For peer review only

| | | | | | | | | | | |
|---|---|---|---|---|---|---|---|---|---|---|
| Biyowale Health Center | -0.150 | 0.00 | 0.122 | 0.36 | 0.132 | 0.01 | 0.335 | 0.00 | 0.171 | 0.23 |
| Legeharae Health Center | 0.114 | 0.01 | 0.188 | 0.00 | 0.116 | 0.00 | 0.129 | 0.06 | 0.122 | 0.01 |
| Melka Jebdu Health Center | 0.104 | 0.08 | 0.173 | 0.02 | 0.106 | 0.00 | 0.089 | 0.11 | 0.101 | 0.15 |
| Wahil Health Center | 0.078 | 0.09 | -0.157 | 0.00 | -0.002 | 0.94 | -0.272 | 0.00 | 0.109 | 0.00 |
| Gende Gerada Health Center | 0.085 | 0.16 | 0.122 | 0.00 | 0.076 | 0.24 | 0.217 | 0.23 | 0.004 | 0.97 |
| Goro Health Center | 0.152 | 0.00 | 0.344 | 0.00 | 0.050 | 0.15 | 0.178 | 0.01 | -0.003 | 0.96 |
| Jelobelina Health Center | 0.133 | 0.07 | 0.243 | 0.00 | 0.158 | 0.01 | 0.160 | 0.05 | 0.106 | 0.04 |
| Constant | 0.470 | 0.00 | 0.380 | 0.00 | 0.729 | 0.00 | 0.778 | 0.00 | 0.063 | 0.34 |
| N obs | 454 | | 440 | | 453 | | 387 | | 455 | |

For peer review only

**Appendix 9.** Comparison of average vs best coworker performance for deliveries with 3 or more providers

| | Coworkers' average performance | | Best coworker's performance | |
|---|---|---|---|---|
| | Coef. | p value | Coef. | p value |
| Group characteristics | | | | |
| Coworkers' average performance across all deliveries | 0.045 | 0.01 | | |
| Best coworker's performance across all deliveries | | | 0.023 | 0.08 |
| Coworkers are more senior cadre than index | -0.054 | 0.04 | -0.062 | 0.02 |
| Coworkers are more experienced than index | -0.024 | 0.27 | -0.023 | 0.29 |
| Number of providers | 0.008 | 0.68 | 0.003 | 0.88 |
| Index provider characteristics | | | | |
| Years of experience | -0.005 | 0.25 | -0.004 | 0.29 |
| Provider cadre (Midwife or nurse diploma ref.) | | | | |
| Midwife or nurse Bsc. | -0.053 | 0.05 | -0.06 | 0.04 |
| Health officer | -0.173 | 0.34 | -0.181 | 0.33 |
| GP | -0.088 | 0.02 | -0.093 | 0.02 |
| IESO | 0.001 | 0.99 | -0.004 | 0.97 |
| Context and environment | | | | |
| Delivery complication | 0.015 | 0.52 | 0.017 | 0.47 |
| Higher risk pregnancy | 0.018 | 0.57 | 0.018 | 0.58 |
| Night delivery (morning reference) | 0.052 | 0.01 | 0.052 | 0.01 |
| Client wealth index (poorest reference) | | | | |
| Wealth 2 | -0.028 | 0.40 | -0.03 | 0.37 |
| Wealth 3 | 0.022 | 0.47 | 0.017 | 0.56 |
| Wealth 4 | -0.019 | 0.57 | -0.023 | 0.50 |
| Wealth 5 (wealthiest) | -0.015 | 0.63 | -0.019 | 0.55 |
| Delivery stage (first exam ref.) | | | | |
| First stage of labor | -0.01 | 0.71 | -0.011 | 0.68 |
| Third stage of labor | 0.208 | 0.00 | 0.208 | 0.00 |
| Immediate newborn care | 0.247 | 0.00 | 0.246 | 0.00 |
| Immediate postpartum care | -0.335 | 0.00 | -0.335 | 0.00 |
| Facility (Dil Chorra Hospital ref.) | | | | |
| Sabien Primary Hospital | 0.034 | 0.23 | 0.044 | 0.12 |
| Legeharae Health Center | 0.052 | 0.45 | 0.102 | 0.11 |
| Gende Gerada Health Center | 0.139 | 0.16 | 0.162 | 0.10 |
| Goro Health Center | 0.1 | 0.09 | 0.141 | 0.01 |
| Constant | 0.453 | 0.00 | 0.46 | 0.00 |
| N | 615 | | 615 | |

For peer review only

**Appendix 10.** Association between coworker performance and quality by provider cadre rank

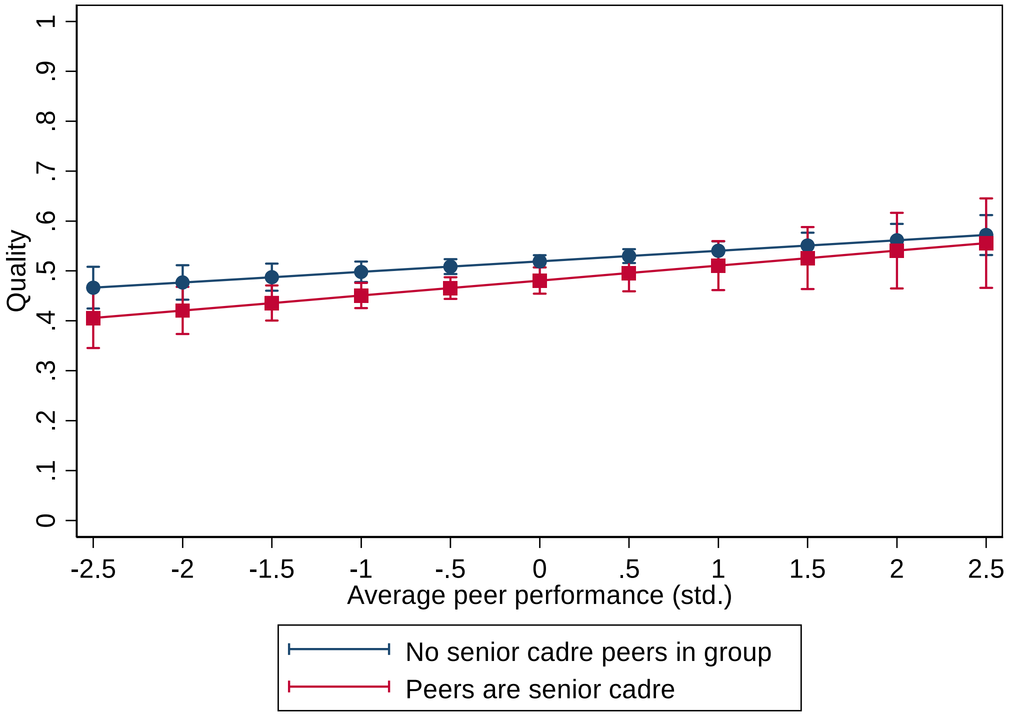


**Appendix 11**. Association between coworker performance and quality by provider seniority (years of experience)

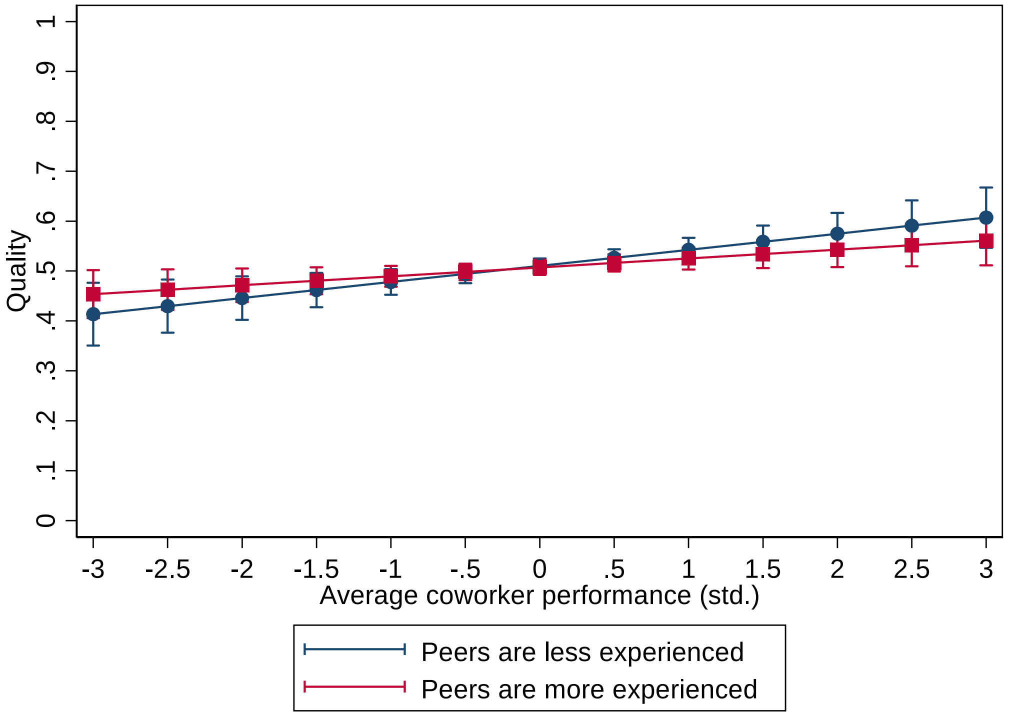

STROBE Statement—Checklist of items that should be included in reports of ***cross-sectional studies***

| | Item No | Recommendation | Page No |
|---|---|---|---|
| **Title and abstract** | 1 | (*a*) Indicate the study's design with a commonly used term in the title or the abstract | 1 |
| | | (*b*) Provide in the abstract an informative and balanced summary of what was done and what was found | 1 |
| **Introduction** | | | |
| Background/rationale | 2 | Explain the scientific background and rationale for the investigation being reported | 2 |
| Objectives | 3 | State specific objectives, including any prespecified hypotheses | 2 |
| **Methods** | | | |
| Study design | 4 | Present key elements of study design early in the paper | 2 |
| Setting | 5 | Describe the setting, locations, and relevant dates, including periods of recruitment, exposure, follow-up, and data collection | 3,4 |
| Participants | 6 | (*a*) Give the eligibility criteria, and the sources and methods of selection of participants | 4 |
| Variables | 7 | Clearly define all outcomes, exposures, predictors, potential confounders, and effect modifiers. Give diagnostic criteria, if applicable | 4-7 |
| Data sources/ measurement | 8* | For each variable of interest, give sources of data and details of methods of assessment (measurement). Describe comparability of assessment methods if there is more than one group | *4-7* |
| Bias | 9 | Describe any efforts to address potential sources of bias | 7 |
| Study size | 10 | Explain how the study size was arrived at | |
| Quantitative variables | 11 | Explain how quantitative variables were handled in the analyses. If applicable, describe which groupings were chosen and why | 4-7 |
| Statistical methods | 12 | (*a*) Describe all statistical methods, including those used to control for confounding | 7 |
| | | (*b*) Describe any methods used to examine subgroups and interactions | 7 |
| | | (*c*) Explain how missing data were addressed | 7 |
| | | (*d*) If applicable, describe analytical methods taking account of sampling strategy | 7 |
| | | (*e*) Describe any sensitivity analyses | 5,7 |
| **Results** | | | |
| Participants | 13* | (a) Report numbers of individuals at each stage of study—eg numbers potentially eligible, examined for eligibility, confirmed eligible, included in the study, completing follow-up, and analysed | 7 |
| | | (b) Give reasons for non-participation at each stage | 7 |
| | | (c) Consider use of a flow diagram | |
| Descriptive data | 14* | (a) Give characteristics of study participants (eg demographic, clinical, social) and information on exposures and potential confounders | Table 2 |
| | | (b) Indicate number of participants with missing data for each variable of interest | Table 2 |



For peer review only - http://bmjopen.bmj.com/site/about/guidelines.xhtml

| | | | |
|---|---|---|---|
| Outcome data | 15* | Report numbers of outcome events or summary measures | Table 1 and Figure 1 |
| Main results | 16 | (*a*) Give unadjusted estimates and, if applicable, confounder-adjusted estimates and their precision (eg, 95% confidence interval). Make clear which confounders were adjusted for and why they were included | Table 3 and Appendix |
| | | (*b*) Report category boundaries when continuous variables were categorized | N/A |
| | | (*c*) If relevant, consider translating estimates of relative risk into absolute risk for a meaningful time period | 9 |
| Other analyses | 17 | Report other analyses done—eg analyses of subgroups and interactions, and sensitivity analyses | Figure 2 |
| **Discussion** | | | |
| Key results | 18 | Summarise key results with reference to study objectives | 11 |
| Limitations | 19 | Discuss limitations of the study, taking into account sources of potential bias or imprecision. Discuss both direction and magnitude of any potential bias | 12 |
| Interpretation | 20 | Give a cautious overall interpretation of results considering objectives, limitations, multiplicity of analyses, results from similar studies, and other relevant evidence | 11 |
| Generalisability | 21 | Discuss the generalisability (external validity) of the study results | 12 |
| **Other information** | | | |
| Funding | 22 | Give the source of funding and the role of the funders for the present study and, if applicable, for the original study on which the present article is based | submission |

*Give information separately for exposed and unexposed groups.

**Note:** An Explanation and Elaboration article discusses each checklist item and gives methodological background and published examples of transparent reporting. The STROBE checklist is best used in conjunction with this article (freely available on the Web sites of PLoS Medicine at http://www.plosmedicine.org/, Annals of Internal Medicine at http://www.annals.org/, and Epidemiology at http://www.epidem.com/). Information on the STROBE Initiative is available at www.strobe-statement.org.

For peer review only

BMJ Open

# The role of teams in shaping quality of obstetric care: a cross-sectional study in Dire Dawa, Ethiopia

| | |
|---|---|
| Journal: | *BMJ Open* |
| Manuscript ID | bmjopen-2022-066111.R1 |
| Article Type: | Original research |
| Date Submitted by the Author: | 16-Sep-2022 |
| Complete List of Authors: | Gage, Anna; Harvard University T H Chan School of Public Health, Global Health and Population; Institute for Health Metrics and Evaluation<br>Yakob, Bereket; The University of British Columbia School of Population and Public Health, Fenot Project; Wolaita Sodo University College<br>McConnell, Margaret; Harvard University T H Chan School of Public Health, Global Health and Population<br>Girma, Tsinuel; The University of British Columbia School of Population and Public Health, Fenot Project<br>Damtachew, Brook; Dil Chora Hospital, Department of Obstetrics and Gynecology<br>Bauhoff, Sebastian; Harvard University T H Chan School of Public Health; Inter-American Development Bank<br>Kruk, Margaret; Harvard University T H Chan School of Public Health, Global Health and Population |
| <b>Primary Subject Heading</b>: | Obstetrics and gynaecology |
| Secondary Subject Heading: | Health services research |
| Keywords: | OBSTETRICS, Human resource management < HEALTH SERVICES ADMINISTRATION & MANAGEMENT, Quality in health care < HEALTH SERVICES ADMINISTRATION & MANAGEMENT |
| | |

SCHOLARONE™
Manuscripts

For peer review only - http://bmjopen.bmj.com/site/about/guidelines.xhtml

*I, the Submitting Author has the right to grant and does grant on behalf of all authors of the Work (as defined in the below author licence), an exclusive licence and/or a non-exclusive licence for contributions from authors who are: i) UK Crown employees; ii) where BMJ has agreed a CC-BY licence shall apply, and/or iii) in accordance with the terms applicable for US Federal Government officers or employees acting as part of their official duties; on a worldwide, perpetual, irrevocable, royalty-free basis to BMJ Publishing Group Ltd ("BMJ") its licensees and where the relevant Journal is co-owned by BMJ to the co-owners of the Journal, to publish the Work in this journal and any other BMJ products and to exploit all rights, as set out in our licence.*

*The Submitting Author accepts and understands that any supply made under these terms is made by BMJ to the Submitting Author unless you are acting as an employee on behalf of your employer or a postgraduate student of an affiliated institution which is paying any applicable article publishing charge ("APC") for Open Access articles. Where the Submitting Author wishes to make the Work available on an Open Access basis (and intends to pay the relevant APC), the terms of reuse of such Open Access shall be governed by a Creative Commons licence – details of these licences and which Creative Commons licence will apply to this Work are set out in our licence referred to above.*

*Other than as permitted in any relevant BMJ Author's Self Archiving Policies, I confirm this Work has not been accepted for publication elsewhere, is not being considered for publication elsewhere and does not duplicate material already published. I confirm all authors consent to publication of this Work and authorise the granting of this licence.*

For peer review only

# The role of teams in shaping quality of obstetric care: a cross-sectional study in Dire Dawa, Ethiopia

Anna D. Gage[1], Bereket Yakob[2,3], Margaret McConnell[1], Tsinuel Girma Nigatu[2], Brook Damtachew[4], Sebastian Bauhoff[1,5], Margaret Kruk[1]

[1]Harvard T.H. Chan School of Public Health, Department of Global Health and Population, Boston, United States
[2]University of British Columbia School of Population and Public Health, Fenot Project, Addis Ababa, Ethiopia
3Woliata Sodo University, School of Public Health, College of Health Sciences and Medicine, Sodo, Ethiopia
[4]Dil Chora Hospital, Department of Obstetrics and Gynecology, Dire Dawa, Ethiopia
[5]Inter-American Development Bank

Corresponding author:
Anna Gage
665 Huntington Ave, Building 1, 11th Floor
Boston, MA 02115
United States
agage@hsph.harvard.edu

For peer review only

## Abstract

Objectives: To examine how characteristics of clinical colleagues influence quality of care.

Design: We conducted a cross-sectional observational study examining the associations between quality of care and a provider's coworkers, controlling for individual provider's characteristics and contextual factors.

Setting: Nine health facilities in Dire Dawa Administration, Ethiopia from December 2020-February 2021.

Participants: 824 clients and 95 unique providers were observed across the nine health facilities.

Outcome measures: We examine the quality of processes of intrapartum and immediate postpartum care during five phases of the delivery (first exam, first stage of labor, third stage of labor, immediate newborn care, and immediate maternal postpartum care).

Results: For the average client, 50% of the recommended routine clinical actions were completed during the delivery overall, with immediate maternal postpartum care being the least well performed (17% of recommended actions). Multiple healthcare providers were involved in 55% of deliveries. The number of providers contributing to a delivery was unassociated with the quality of care, but a one standard deviation increase in the coworker's performance was associated with a two-percentage point increase in quality of care ($p<0.01$); this association was largest among providers in the middle quartiles of performance.



For peer review only - http://bmjopen.bmj.com/site/about/guidelines.xhtml

Conclusions: A provider's typical performance had a modest positive association with quality of delivery care given by their coworker. As delivery care is often provided by multiple healthcare providers, examining the dynamics of how they influence one another can provide important insights for quality improvement.

**Strengths and limitations of this study**

- This is among the first studies to examine how multiple providers influence one another to provide quality of care in a low-income healthcare setting.
- We use detailed observations of quality of care with actions attributed to specific providers.
- The study examines cross-sectional associations and the effects cannot be interpreted causally.
- We examine quality of routine deliveries only; further research should examine the role of provider groups in managing complications.

For peer review only



For peer review only - http://bmjopen.bmj.com/site/about/guidelines.xhtml

## INTRODUCTION

Ethiopia's health system, like those in other low-resource settings, has gaps in maternal and newborn care quality that pose a barrier to improved health outcomes. Although the facility delivery rate has increased dramatically in the past 10 years from 10% in 2011 to 48% in 2019, maternal and newborn mortalities remain high, with 401 maternal deaths per 100,000 live births and 33 neonatal deaths per 1000 live births.[1,2] The majority of such deaths could be avoided with high quality neonatal and maternal care.[3,4] However, in 2018, just 20% of mothers who delivered in a health facility were estimated to receive a high quality of routine maternal care in the Tigray region in northern Ethiopia.[5]

Examinations of poor maternal care quality in similar contexts have found that there is often wide variation in the quality that a single provider will provide across different deliveries. For example, in Uganda the quality of routine actions during labor and delivery varied as much as sixty percentage points across the deliveries for which a single healthcare provider was responsible.[6] In Kenya and Malawi, two studies found that the provider contributed very little to the explained variance in the technical and respectful quality of maternity care.[7,8] The importance of the provider to quality may vary over the course of a delivery, however: another study found that healthcare providers were more important in accounting for the quality of the intrapartum period rather than the assessment or postpartum phases.[9] While these studies have also found that facility, region, and country-level factors also play important roles in determining the quality of care, it is worth further considering why provider quality varies in order to shed light on potential improvement strategies.

One limitation of the above studies in their examination of quality is the focus on attribution of quality to a single healthcare provider over the course of a labor and delivery. While many mothers in Ethiopia receive care from a single healthcare provider, this is not the only model. Rather, multiple healthcare providers may attend a single delivery over the course of the labor, delivery and immediate postpartum period, particularly during more complex cases that lead to higher morbidity and mortality. When groups of providers attend a single patient, their interactions with one another may affect the quality of care.[10] Indeed, the disciplines of complexity science and team science suggest that provider groups are more than the collection of independently acting individuals, but rather they may influence one another in nonlinear and dynamic ways.[10,11] The culmination of these interactions between providers creates the informal group norms and culture around the quality of care that may affect realized quality and health outcomes.

Groups or teams of providers have been examined frequently in healthcare contexts in high-income countries. For example, studies have found that the quality of HIV care is influenced by the performance of a provider's peers on quality[12] and that a provider's patterns of prescribing medications are affected by a specialist in a provider's network.[13] Similarly, studies have found moderate effects of group characteristics such as professional composition and aspects of teamwork such as coordination on quality of care.[14–16] However, these dynamics have not been examined in healthcare in low- and middle-income countries. Using cross-sectional observations of delivery care in Ethiopia and adopting a complex systems lens, this study seeks to understand



For peer review only - http://bmjopen.bmj.com/site/about/guidelines.xhtml

how the number of providers and group dynamics are associated with the quality of delivery care.

# METHODS

## Conceptual framework

We draw on the theory of small groups as complex systems to inform the conceptual framework for this analysis.[11] In this analysis, we define a group of healthcare providers to be all providers who care for a specific woman and newborn over the course of the delivery. Their group identity is thus focused on the shared objective of providing high quality care, and they draw on one another as well as the broader facility environment in achieving this objective.

Arrow et al argue that group behavior involves interactions at three levels.[11] First, behavior is influenced by the constituent elements of the groups, in this case the individual providers who bring different experiences, training, and roles to the group. Second, there are interactions of the group as an entity, which will include feedback loops between group members. Third, there are interactions with the context in which the group is embedded; this context includes the characteristics of the facility as well as the characteristics of the specific delivery for which the group is providing care.

We focused our analysis on the interactions of the group as an entity, with an emphasis on potential spillovers on quality of care. We also account for care context. Drawing on the peer effects literature, we hypothesize several mechanisms by which group members may influence one another in their provision of quality care. First, members may influence one another through hierarchal relationships defined between providers by their cadre rank or years of experience. For example, a provider working with their superior may perform better due to their better supervision or social pressure. Second, there may be informal influence between providers of different abilities. When working with a high performing group member, there may be positive spillovers because of social pressure, knowledge spillovers or social comparison.[17] Conversely, there may negative spillovers due to free riding.[17] While our data does not permit investigation into these specific mechanisms, we assessed whether working with a high performing colleague is associated with higher or lower performance.

## Setting

Dire Dawa is a city administration in Eastern Ethiopia with a population in 2019 of approximately 493,000 people, with 63% living in urban Dire Dawa City and the remaining in rural areas surrounding the City.[18] In the five years preceding 2019, 84% of pregnant women received at least one antenatal care visit and 69% delivered in a health facility.[18] Despite rising utilization rates, neonatal mortality increased between 2011 and 2016 to 36 deaths per 1000 live births.[1] In 2021, the administration had 53 public health facilities: 2 hospitals, 15 health centers and 35 health posts. In addition, there were 5 private hospitals and 7 private clinics.

## Data


For peer review only - http://bmjopen.bmj.com/site/about/guidelines.xhtml

This analysis is part of a broader study to understand the quality of maternal and immediate postpartum care in Dire Dawa Administration's public health system. Cross-sectional primary data was collected in 9 facilities. Public facilities with the highest volumes of deliveries using the 2019 health information system data were included in the study; these facilities were collectively responsible for 83% of the facility deliveries in the region in 2019. The selected facilities consisted of two public hospitals, four health centers in Dire Dawa City and three rural health centers.

Data tools relevant to this analysis include observations of deliveries, interviews with observed clients upon discharge from the facility, and interviews with all providers who provided care. First, all providers who provided intrapartum or immediate postpartum care in the study facilities were invited to take part in a provider survey that asked about their training, perceptions of the working environment and quality of care, and knowledge of complications diagnoses and management.

Second, quality of care was assessed through observations of deliveries by trained health workers. All clients presenting for delivery during the observation period were invited to participate in the study and their care was observed from the time of arrival at the facility until 6 hours postpartum or discharge from the facility. Data collectors identified which items providers completed on a checklist adapted from the Maternal and Child Health Integrated Program tool from USAID. For groups of actions (i.e. first exam), the collector indicated which provider conducted the actions. For this analysis, only deliveries that were observed from admission to discharge were included, so Cesarean section deliveries and intrapartum referrals were excluded. The observation checklist was organized into discrete modules; the ones relevant to this analysis are those for the first exam, checks on the client during the first stage of labor, third stage of labor, immediate newborn care and immediate maternal postpartum care. Finally, all participants whose care was observed were invited to participate in an exit interview upon their discharge from the facility.

Observations of care were conducted in most facilities from December 22, 2020 to February 20, 2021. However, until January 31, 2021, one of the hospitals (Sabian Primary Hospital) was not accepting maternity patients because it was a designated Covid-19 treatment facility. Observations of delivery care in Sabian, therefore, occurred between February 13 and March 21, 2021. Ethical approval for this study was obtained from Harvard University IRB (IRB19-0926), Haramaya University IRB (IHRERC/138/219) and the Ethiopia National Research Ethics and Review Committee (MoSHE//RD/1411/9403/RO).

**Outcome**

The primary outcome of interest for this analysis was the quality of routine maternal care, adapted from the quality of processes of intrapartum and immediate postpartum care index (QoPIIPC).[19] The original index contains 20 indicators of routine actions that should be conducted during every delivery. To attribute the performance of the indicators to a particular provider, we separated this index into five sub-indices associated with the module of the checklist (first exam, first stage of labor, third stage of labor, immediate newborn care and immediate maternal postpartum care). For example, the first exam sub-index consists of seven



For peer review only - http://bmjopen.bmj.com/site/about/guidelines.xhtml

items that should be completed during the first exam such as taking pulse and asking whether the client experienced vaginal bleeding. Index items are presented in Table 1. Performance on each sub-index, which we refer to as the quality of a stage, is calculated as the percent of actions that were completed so it ranges from 0 to 100%. We attributed the quality of each stage for every delivery to the provider who was noted as providing care during that stage. Deliveries with multiple providers had differences in how stages were assigned, see Appendix 1 for an example of this structure.

**Table 1**. Components of quality of intrapartum and immediate postpartum care processes index by delivery stage

| | Percent complete | N |
|---|---|---|
| Average of first exam actions | 47% | 823 |
| Checks woman's HIV status | 68% | 809 |
| Asks whether woman has experienced headaches or blurred vision | 6% | 823 |
| Asks whether woman has experienced vaginal bleeding | 7% | 823 |
| Takes blood pressure during initial client assessment | 73% | 822 |
| Takes pulse during initial client assessment | 67% | 822 |
| Washes hands before initial examination | 13% | 823 |
| Wears gloves before vaginal examination | 99% | 781 |
| Average of first stage of labor actions | 48% | 795 |
| At least once, explains what will happen in labor | 42% | 824 |
| Prepares uterotonic drug to use for AMTSL | 81% | 791 |
| Uses partograph during labor | 53% | 793 |
| Prepares bags and masks for neonatal resuscitation | 14% | 783 |
| Average of third stage of labor actions | 72% | 822 |
| Correctly administers uterotonic | 56% | 820 |
| Assesses completeness of placenta and membranes | 74% | 819 |
| Assesses for perineal and vaginal membranes | 90% | 819 |
| Ties or clamps cord when pulsations stop, or by 2-3 minutes after birth | 54% | 821 |
| Average of immediate newborn care items | 81% | 694 |
| Immediately dries baby with towel | 98% | 694 |
| Places newborn on mother's abdomen skin-to-skin | 64% | 690 |
| Average of immediate maternal postpartum actions | 17% | 824 |
| Takes mother's vital signs 15 minutes after birth | 0.2% | 823 |

For peer review only


For peer review only - http://bmjopen.bmj.com/site/about/guidelines.xhtml

| | | |
|---|---|---|
| Palpates uterus 15 minutes after birth | 19% | 824 |
| Assists mother to initiate breastfeeding | 37% | 695 |
| Quality of Intrapartum and Immediate Postpartum Care Processes index | 51% | 828 |

Notes: AMTSL: Active management of the third stage of labor

Several of the indicators included a timeliness component, for example, whether the provider checked the mother's vital signs 15 minutes after birth. To credit providers who completed these actions outside of the designated timeframe, we also constructed an alternate index without any time limits on the actions as a sensitivity analysis.

13% of delivery observations did not include the module on checks during the first stage of labor. We deemed 30% of these missing observations as "valid missing" if labor was induced directly after the first exam or if there was less than 60 minutes between the end of the first exam and delivery, as there may not have been time for the enumerator to complete the module. For the remaining observations, non-performance of the actions under the first stage (e.g. missing actions imputed as 0) was attributed to the provider who conducted the first exam. Among observations where this stage was not missing, the same provider conducted the first exam and the first stage 65% of the time, while 33% of observations had the same provider for the first and third stages of labor.

**Independent variables**

As described above, each stage of a delivery was assigned to a single responsible provider. We defined a group as the providers that cared for a single client at different stages of her delivery. For deliveries with more than one provider, we were interested in how quality for a particular delivery stage completed by the index provider is associated with the characteristics of other providers in the group. While there may be many other providers working at a facility that are unassociated with a delivery, we define these specific provider groups as the index provider's coworkers for the delivery.

We defined three independent variables of interest. First, we examined the cadre of the coworkers relative to the index provider. We created a binary variable for whether the coworkers had a cadre superior to the index provider. There are five categories of cadres, here ranked from low to high: midwife or nurse (diploma), midwife or nurse (Bsc.), health officer, general practitioner, and integrated emergency surgical officer (IESO). Second, we examined the years of professional experience of the coworkers relative to the index provider. Similar to cadre, we created a binary variable for whether coworkers were more experienced than the index provider.

Third, we defined a measure of coworker performance following a two-step approach.[17] In the first step, we created an individual provider performance score for every provider observed, which captures the index provider's own capabilities. We specified a fixed effects model to estimate the provider's ability across all deliveries and delivery stages where they were the responsible provider. We controlled for delivery stage because of the differences in quality between each stage. The provider's fixed effect became a measure of an individual provider's capabilities.


For peer review only - http://bmjopen.bmj.com/site/about/guidelines.xhtml

In the second step, we took the average of the provider capabilities measure for all other providers who cared for the same client besides the index provider. As a sensitivity analysis for deliveries with three or more providers, we also compared the average coworker performance with the best coworker's performance. The coworker performance measure was standardized for the analysis.

**Covariates**

Drawing on the conceptual framework, we defined several covariates for the individual providers and the contextual environment. First, we included the index provider's cadre and number of years of experience, drawn from the provider interviews. Second, the context environment included characteristics of the client, the delivery, and the facility. These include whether the birth had a complication (neonatal resuscitation initiated, newborn referred to the NICU, or mother treated for post-partum hemorrhage or eclampsia); whether the delivery was at higher risk for a complication (grand multiparity, mother younger than 18 or older than 35, or multiple births); time of delivery (morning 8 am-6 pm, or night 6 pm-8 am); and the client's wealth (defined by quintiles within the analytic sample using exit interview asset index). Finally, we also controlled for delivery stage as defined above and facility fixed effects.

**Analysis**

We first described the quality of care provided to the study sample on the QoPIIPC index and the characteristics of the clients and providers. We examined whether quality differed by the number of providers caring for the client and examined what factors were associated with group-based care.

We fit a linear mixed-effects model to assess the contribution of group dynamics to quality of care, with observations of delivery stages nested within the index provider. Standard errors were clustered at the index provider. Covariates were missing for a small number of providers and clients; we used multiple imputation such that all observed deliveries meeting the criteria could be included in the analysis. This analysis was conducted among all group deliveries. In addition, we conducted sub-analyses separately for each stage of delivery.

We further investigated how the coworker performance measure interacts with the other group characteristics. First, we categorized the index provider's performance into quintiles and interacted it with coworker performance to understand how relative performance may be associated with quality. Second, we also ran interaction models between coworker performance and the two measures of provider rank (cadre and years of experience) respectively. We used these interaction models to predict quality across the range of coworker performance holding all other covariates at their means and graphed these marginal models.

**Patient and public involvement**

Patients were not involved in the design or implementation of this study.



For peer review only - http://bmjopen.bmj.com/site/about/guidelines.xhtml

# RESULTS

Over the data collection period, 983 clients in the nine facilities were invited to participate in the study. 979 clients (99.6%) agreed to participate, and 828 (84%) were observed throughout the whole delivery and thus met the inclusion criteria for this analysis. The observed clients were cared for by 95 unique providers; 84 (88%) of them were interviewed for the study.

Client and provider characteristics are shown in Table 2. 452 (54%) of the observed deliveries took place at one of the two study hospitals, while the remaining deliveries were at the seven health centers. 213 (26%) of clients experienced a complication during the delivery and 87 (11%) had a higher risk pregnancy. 457 (55%) of deliveries had more than one provider attending over the course of their delivery: 72% of hospital deliveries and 35% of health center deliveries. Among the deliveries with more than one provider, the mean number of providers was 2.4. Two thirds of providers were midwives or nurses (BSc.); providers had an average of 6.2 years of cumulative professional experience.

**Table 2**. Delivery and provider characteristics

| | All deliveries | | Hospitals (N=2) | | Health centers (N=7) | |
|---|---|---|---|---|---|---|
| | N | % | N | % | N | % |
| Client and delivery characteristics | | | | | | |
| N deliveries observed | 828 | | 452 | | 376 | |
| Client's primary language | | | | | | |
| Oromiffa | 484 | 63% | 196 | 49% | 288 | 79% |
| Amharic | 178 | 23% | 129 | 32% | 49 | 13% |
| Somali | 81 | 11% | 52 | 13% | 29 | 8% |
| Other | 23 | 3% | 23 | 6% | 0 | 0% |
| Poorest wealth quintile | 150 | 18% | 52 | 12% | 98 | 26% |
| Experienced complication | 213 | 26% | 69 | 15% | 144 | 38% |
| Higher risk pregnancy | 87 | 11% | 45 | 1% | 42 | 11% |
| Time of delivery | | | | | | |
| Day (8 am-6 pm) | 341 | 41% | 186 | 41% | 155 | 41% |
| Night (6 pm-8 am) | 487 | 59% | 266 | 59% | 221 | 59% |
| Provider characteristics | | | | | | |
| N providers interviewed | 84 | | 50 | | 34 | |
| Years of experience (mean/sd) | 6.2 | 4.5 | 5.9 | 3.6 | 6.5 | 5.7 |
| Female | 54 | 64% | 31 | 62% | 23 | 68% |



For peer review only - http://bmjopen.bmj.com/site/about/guidelines.xhtml

| | | | | | | |
|---|---|---|---|---|---|---|
| Cadre | | | | | | |
| Midwife or Nurse (diploma) | 13 | 15% | 7 | 14% | 6 | 18% |
| Midwife or nurse (BSc) | 55 | 65% | 33 | 66% | 22 | 65% |
| Health officer | 3 | 4% | 0 | 0% | 3 | 9% |
| General practitioner | 7 | 8% | 4 | 8% | 3 | 9% |
| IESO | 6 | 7% | 6 | 12% | 0 | 0% |
| Provider group characteristics | | | | | | |
| Deliveries with >1 provider | 457 | 55% | 326 | 72% | 131 | 35% |
| N providers among group deliveries (mean/sd) | 2.38 | 0.63 | 2.48 | 0.70 | 2.12 | 0.35 |
| At least one health officer, GP or IESO among providers in group | 76 | 16% | 65 | 20% | 11 | 8% |
| At least one provider with over median years of experience | 198 | 43% | 119 | 37% | 79 | 60% |

Notes: Delivery complication is neonatal resuscitation, newborn referred to the NICU, or mother treated for post-partum hemorrhage or eclampsia. Higher risk is mother grand multiparous (5 or more births), younger than 18 or older than 35, or has multiple births. IESO: Integrated Emergency Surgical Officers.

The quality of intrapartum and immediate postpartum care processes provided to the clients in this study was poor (Table 1). During the average delivery, only half of the recommended routine actions were done. Actions ranged from 0.2% of mothers whose vitals were checked fifteen minutes after birth to 98% of babies that were dried immediately with a towel. The recommended actions were most often completed for the immediate newborn care stage, while they were least often done during the immediate maternal postpartum care stage. When the index was defined without any time constraints on the actions, average quality of care rose to 58% of recommended actions across the whole delivery (Appendix 2). However, still only 3% of mothers had their vitals checked after delivery. Over 90% of the variance in quality of care was at the client level, rather than the facility or index provider levels (Appendix 3).

Between 1 and 5 providers cared for a single client and baby over the course of the delivery. The number of providers was unassociated with quality of care (Figure 1 and Appendix 4). The primary determinant of having more than one provider care for a client (Appendix 5) was the hospital delivery. Time of delivery, client wealth and language were slightly associated with a group delivery, but neither higher risk clients nor complicated deliveries was associated with more providers. The remaining results are only among deliveries with more than one provider.

In the bivariate associations between group characteristics and quality of delivery stages (Appendix 6), coworkers' performance and seniority by cadre are associated with quality of care. Scatterplots of the index provider and coworker performance (Appendix 7) further show that this is in part due to quality clustering by facility. Table 3 shows the associations of coworker



For peer review only - http://bmjopen.bmj.com/site/about/guidelines.xhtml

characteristics with quality of care adjusted for all covariates. After controlling for the facility fixed effect, index provider and client characteristics, a one standard deviation increase in coworker performance was associated with a 2-percentage point increase in quality of care during a given delivery stage, or 4% relative to the mean performance. This association was larger during the first stage of labor (4.2 percentage point increase) (Appendix 8). Working with coworkers that were superior in rank to the index provider was associated with 3.5 percentage point lower quality, while the coworker's relative experience was unassociated with quality. In deliveries with three or more providers, the average coworker performance had a higher association with quality than the maximum coworker performance (Appendix 9).

**Table 3.** Group dynamics associations with quality of care (Outcome range 0-1)

| | Quality of care | | |
|---|---|---|---|
| | Coef. | p value | 95% CI |
| Group characteristics | | | |
| Peers' performance across all deliveries | 0.023 | 0.00 | (0.01,0.04) |
| Peers are more senior cadre than index | -0.035 | 0.02 | (-0.07,-0.01) |
| Peers are more experienced than index | -0.009 | 0.46 | (-0.03,0.02) |
| Number of providers | 0.000 | 0.96 | (-0.02,0.02) |
| Index provider characteristics | | | |
| Years of experience | -0.002 | 0.41 | (-0.01,0.00) |
| Provider cadre (Midwife or nurse diploma ref.) | | | |
| Midwife or nurse Bsc. | -0.043 | 0.04 | (-0.09,-0.00) |
| Health officer | -0.141 | 0.02 | (-0.25,-0.03) |
| GP | -0.064 | 0.00 | (-0.11,-0.02) |
| IESO | -0.066 | 0.48 | (-0.25,0.12) |
| Context and environment | | | |
| Delivery complication | 0.004 | 0.81 | (-0.03,0.04) |
| Higher risk pregnancy | 0.003 | 0.88 | (-0.04,0.04) |
| Night delivery (morning reference) | 0.007 | 0.51 | (-0.01,0.03) |
| Client wealth index (poorest reference) | | | |
| Wealth 2 | 0.037 | 0.06 | (-0.00,0.07) |
| Wealth 3 | 0.071 | 0.00 | (0.03,0.11) |
| Wealth 4 | 0.039 | 0.05 | (-0.00,0.08) |
| Wealth 5 (wealthiest) | 0.050 | 0.01 | (0.01,0.09) |
| Delivery stage (first exam ref.) | | | |



For peer review only - http://bmjopen.bmj.com/site/about/guidelines.xhtml

| | | | |
|---|---|---|---|
| First stage of labor | 0.001 | 0.96 | (-0.03,0.04) |
| Delivery care | 0.233 | 0.00 | (0.20,0.26) |
| Immediate newborn care | 0.300 | 0.00 | (0.26,0.34) |
| Immediate postpartum care | -0.320 | 0.00 | (-0.35,-0.29) |
| Facility (Dil Chorra Hospital ref.) | | | |
| Sabien Primary Hospital | 0.017 | 0.35 | (-0.02,0.05) |
| Biyowale Health Center | 0.127 | 0.01 | (0.04,0.22) |
| Legeharae Health Center | 0.129 | 0.00 | (0.07,0.19) |
| Melka Jebdu Health Center | 0.117 | 0.00 | (0.06,0.18) |
| Wahil Health Center | -0.049 | 0.09 | (-0.11,0.01) |
| Gende Gerada Health Center | 0.083 | 0.05 | (0.00,0.16) |
| Goro Health Center | 0.148 | 0.00 | (0.09,0.21) |
| Jelobelina Health Center | 0.160 | 0.00 | (0.09,0.23) |
| Constant | 0.438 | 0.00 | (0.35,0.53) |
| N obs | 2189 | | |

Notes: Regressions are at delivery-stage level, with stages nested within providers. Delivery complication is neonatal resuscitation, newborn referred to the NICU, or mother treated for post-partum hemorrhage or eclampsia. Higher risk is mother grand multiparous (5 or more births), younger than 18 or older than 35, or has multiple births

Given that each subindex contains between 3 and 7 items, these coefficients translate to less than one additional action completed. However, the coefficients are large in comparison to the risk characteristics: deliveries that had a complication or were from higher risk pregnancies were not more likely to have these routine actions completed than less risky deliveries. The quality of care that women in the wealthiest quintile received was on average 5 percentage points higher than women in the poorest wealth quintile.

The marginal associations of coworker performance by the index provider's capabilities are shown in Figure 2. There is no association between coworker performance and quality among the top performing providers: they perform consistently well regardless of their coworkers' performance. However, providers in the middle and low quartiles have large improvement when surrounded by better coworkers. For example, the predicted performance of a third quartile provider when their coworkers' performance is one standard deviation above average is 53% in comparison to 48% when their coworkers are 1 standard deviation below average. Graphs of coworker performance by seniority are included in Appendix 10 and 11; there are not substantive differences in the associations by either cadre rank or years of experience relative to the index provider.

**DISCUSSION**



For peer review only - http://bmjopen.bmj.com/site/about/guidelines.xhtml

Deliveries are often attended by multiple providers who must work together to provide high quality care for the mother and newborn, particularly in larger health facilities. Adopting a small groups as complex systems framework, this study examined the provider group dynamics and their associations with quality of care in Dire Dawa, Ethiopia. This study had four key findings. First, the observed quality of routine labor and delivery was poor, especially for postpartum maternal care that is vital in timely diagnosis of potentially fatal conditions such as postpartum hemorrhage. Second, women in the poorest wealth quintiles received worse care, even after controlling for facility and provider characteristics. Third, we found that approximately 70% of hospital deliveries and 35% of health center deliveries had more than one provider involved, but more providers were not associated with higher quality of care. Fourth, providers performed better when working with someone who delivered high quality care, though the differences were small.

We hypothesize several mechanisms by which these provider dynamics may be acting, though our study cannot tease these apart directly. First, we found that if a provider is working with a competent colleague, they do not consequently do less for the client. This is consistent with other peer performance literature, which has shown benefits from working with high-performing colleagues.[12,17] The association with coworker performance was strongest in the first stage of labor, a stage which may require more communication and coordination between providers to share information after the first exam and before delivery. Greater interaction and interdependence between providers during the delivery may therefore create stronger associations. This aligns with recent work demonstrating that increasing the duration and intensity of collaboration between providers can reduce patient mortality.[20] Contrary to our hypothesis, quality of care was lower when coworkers were of a superior cadre to the index provider. Given that the lower cadres provide the routine actions more often than their senior counterparts, there may be some reverse accountability in working with a more diligent junior colleague. Other work has also shown that junior cadres in Ethiopia have smaller gaps between their knowledge and practice.[21]

This work can inform quality improvement in several ways. First, interventions should place an emphasis on improving immediate postpartum maternal care, which has the largest identified gaps and the greatest potential for reducing severe maternal morbidity and mortality. While the gaps in monitoring vitals and palpating the uterus that we found in Dire Dawa are larger than those seen in other areas of Ethiopia, the immediate postpartum period has previously been identified as a neglected area.[22,23] Indeed, in evaluations of quality improvement interventions in Ethiopia, the mother's postpartum care is often not even included as an outcome in favor of immediate newborn care.[24,25] Interventions may emphasize this area in health worker education or regular supervision. Second, the propensity for multiple providers during delivery suggests that health worker education should emphasize team-based models of care, perhaps through team drills for emergency scenarios. Knowing how to work as a team, assign roles and responsibilities and communicate findings may improve the performance of the team.[26] While our study assessed mainly normal births, highly optimized team-based care will also be required to reduce mortality in the case of obstetric emergencies.[27]

Third, improvement interventions may consider management or supervision approaches that pair providers of mixed competency levels. Our analysis shows that only the very top performers do


For peer review only - http://bmjopen.bmj.com/site/about/guidelines.xhtml

not benefit from their coworkers' performance, while most providers may benefit from working with a high performer. Arranging staffing schedules to intentionally pair higher and lower providers may increase the potential for these positive spillovers and strengthen accountability. Supervision approaches typically use traditional cadre hierarchies to define who may act as a supervisor;[28] however, this work suggests that junior cadres may be able to effectively supervise more senior coworkers. Alternately, actual performance could be used to identify potential supervisors.

Finally, the culmination of these interactions between providers over many deliveries creates the facility culture and norms for quality of care. Interventions that aim to change facility norms on quality of care, such as group problem solving or quality improvement collaboratives have shown moderate to high effects on health worker practices in meta-analyses.[29,30] For example, an improvement intervention in India that used an integrated training, mentoring and a plan-do-study-act cycle with an emphasis on peer learning improved postpartum monitoring of mothers from 52% to 94%.[31] However, this study also shows the limits of micro-focused interventions. The potential for a two-percentage point improvement from pairing providers will not overcome the enormous quality deficit seen in labor and delivery care in Dire Dawa. Rather, other macro level strategies such as improved pre-service education or redesigning maternal and newborn care service to be provided at CEmONC capable facilities will likely be necessary.[32,33]

This study is among the first to examine how multiple providers influence one another to provide quality of care in a low-income healthcare setting. The detailed observation data with information on who attended each stage of delivery is a strength of the analysis. There are, however, several limitations to address in future research. First, missing provider attribution for the first stage of delivery required us to make assumptions about who was responsible for the care that was not provided. Second, although we controlled for many potential confounders, the associations may not be interpreted causally. The formation of the provider groups may in some cases be endogenous, for example if a proactive provider actively seeks support from a high-quality provider to assist with a complex delivery. Third, this study only focused on routine actions that should be done for every mother and newborn. However, the quality of complications management is likely both more impactful for consequent health outcomes and often requires multiple providers to work together coincidentally rather than sequentially. Future research should examine the coordination and communications of providers working to address complications. Finally, this study took place during the Covid-19 pandemic in Ethiopia and for a period during the study, one of the two public hospitals in Dira Dawa was closed to deliveries because it was serving as a Covid treatment facility. This resulted in unusually high delivery volumes in the other hospital which may have altered provider dynamics.

## CONCLUSION

In Dire Dawa, Ethiopia, we find that the number of providers attending to a delivery was not associated with quality of delivery, but the characteristics of those providers in relation to one another did potentially impact quality. As more women across Sub-Saharan Africa deliver in hospitals, they are more likely to be attended by multiple healthcare providers, which could have consequences for the quality of care they receive. Unpacking the provider dynamics in how they


For peer review only - http://bmjopen.bmj.com/site/about/guidelines.xhtml

work together to deliver care quality can yield useful insights for quality improvement in the future.

**Author Contributions:** ADG, MK and BY conceived the study. BY, BD and TNG oversaw the data collection. ADG conducted the analysis and wrote the first draft with substantial input from all authors. All authors, ADG, BY, MM, SB, BD, TNG and MK, critically reviewed the draft and approved this version for submission.
**Competing Interests:** None declared.
**Funding:** We acknowledge funding from the Bill & Melinda Gates Foundation [Grant No. OPP1135922].
**Data sharing statement:** Data available from corresponding author upon reasonable request.
**Ethics:** Ethical approval for this study was obtained from Harvard University IRB (IRB19-0926), Haramaya University IRB (IHRERC/138/219) and the Ethiopia National Research Ethics and Review Committee (MoSHE//RD/1411/9403/RO).
**Acknowledgements:** We gratefully acknowledge the contributions from the data collectors and study participants.

For peer review only

## References

1 Ethiopian Public Health Institute. Ethiopia Mini Demographic and Health Survey. Addis Ababa, 2021 https://dhsprogram.com/pubs/pdf/FR363/FR363.pdf (accessed Nov 4, 2021).

2 World Bank. World Development Indicators. 2017. http://databank.worldbank.org/data/reports.aspx?source=World-Development-Indicators# (accessed Feb 10, 2018).

3 Tessema GA, Laurence CO, Melaku YA, *et al.* Trends and causes of maternal mortality in Ethiopia during 1990-2013: findings from the Global Burden of Diseases study 2013. *BMC public health* 2017; **17**: 160.

4 Berhan Y, Berhan A. Perinatal mortality trends in Ethiopia. *Ethiopian journal of health sciences* 2014; **24**: 29–40.

5 Weldearegay HG, Kahsay AB, Medhanyie AA, Godefay H, Petrucka P. Quality of and barriers to routine childbirth care signal functions in primary level facilities of Tigray, Northern Ethiopia: Mixed method study. *PloS one* 2020; **15**: e0234318.

6 Rokicki S, Mwesigwa B, Cohen JL. Know-do gaps in obstetric and newborn care quality in Uganda: a cross-sectional study in rural health facilities. *Tropical Medicine & International Health* 2021; **26**: 535–45.

7 Arsenault C, English M, Gathara D, Malata A, Mandala W, Kruk ME. Variation in competent and respectful delivery care in Kenya and Malawi: a retrospective analysis of national facility surveys. *Tropical Medicine & International Health* 2020; **25**: 442–53.

8 Helfinstein S, Jain M, Ramesh BM, *et al.* Facilities are substantially more influential than care providers in the quality of delivery care received: a variance decomposition and clustering analysis in Kenya, Malawi and India. *BMJ global health* 2020; **5**: e002437.

9 Owili PO, Muga MA, Mendez BR, Chen B. Quality of maternity care and its determinants along the continuum in Kenya: A structural equation modeling analysis. *PLoS One* 2017; **12**: e0177756.

For peer review only

10 Mach M, Abrantes AC, Soler C. Teamwork in Healthcare Management. *Teamwork in Healthcare* 2021; : 23.

11 Arrow H, McGrath JE, Berdahl JL. Small groups as complex systems: Formation, coordination, development, and adaptation. Sage Publications, 2000.

12 Stecher C. Physician network connections to specialists and HIV quality of care. *Health Services Research* 2021.

13 Nair HS, Manchanda P, Bhatia T. Asymmetric social interactions in physician prescription behavior: The role of opinion leaders. *Journal of Marketing Research* 2010; **47**: 883–95.

14 Schmutz JB, Meier LL, Manser T. How effective is teamwork really? The relationship between teamwork and performance in healthcare teams: a systematic review and meta-analysis. *BMJ open* 2019; **9**: e028280.

15 Rosen MA, DiazGranados D, Dietz AS, *et al.* Teamwork in healthcare: Key discoveries enabling safer, high-quality care. *American Psychologist* 2018; **73**: 433.

16 Gharaveis A, Hamilton DK, Pati D. The impact of environmental design on teamwork and communication in healthcare facilities: a systematic literature review. *HERD: Health Environments Research & Design Journal* 2018; **11**: 119–37.

17 Tan TF, Netessine S. When you work with a superman, will you also fly? an empirical study of the impact of coworkers on performance. *Management Science* 2019; **65**: 3495–517.

18 UNICEF. Situation Analysis of Children and Women: Dire Dawa Administration. 2019 https://www.unicef.org/ethiopia/media/2556/file/Dire%20Dawa.pdf (accessed Sept 29, 2021).

19 Tripathi V, Stanton C, Strobino D, Bartlett L. Development and Validation of an Index to Measure the Quality of Facility-Based Labor and Delivery Care Processes in Sub-Saharan Africa. *PLoS One* 2015; **10**: e0129491.

20 Chen Y. Team-specific human capital and team performance: Evidence from doctors. *American Economic Review* 2019.

For peer review only



For peer review only - http://bmjopen.bmj.com/site/about/guidelines.xhtml

21 Gage AD, Kruk ME, Girma T, Lemango ET. The know-do gap in sick child care in Ethiopia. *PLoS One* 2018; **13**: e0208898.

22 Kebede AA, Taye BT, Wondie KY, Tiguh AE, Eriku GA, Mihret MS. Healthcare provider's adherence to immediate postpartum care guidelines in Gondar province hospitals, northwest Ethiopia: A multicenter study. *Plos one* 2021; **16**: e0259263.

23 Fisseha G, Berhane Y, Worku A, Terefe W. Quality of the delivery services in health facilities in Northern Ethiopia. *BMC health services research* 2017; **17**: 1–7.

24 Magge H, Kiflie A, Nimako K, *et al.* The Ethiopia healthcare quality initiative: design and initial lessons learned. *International Journal for Quality in Health Care* 2019; **31**: G180–6.

25 Hagaman AK, Singh K, Abate M, *et al.* The impacts of quality improvement on maternal and newborn health: preliminary findings from a health system integrated intervention in four Ethiopian regions. *BMC health services research* 2020; **20**: 1–12.

26 Fransen AF, de Ven J, Banga FR, Mol BWJ, Oei SG. Multi-professional simulation-based team training in obstetric emergencies for improving patient outcomes and trainees' performance. *Cochrane Database of Systematic Reviews* 2020.

27 Dalby P, Gosman GG, Stein K, Streitman D, Wise N. Crisis teams for obstetric patients. *Textbook of Rapid Response Systems* 2017; : 229–39.

28 Austin A, Gulema H, Belizan M, *et al.* Barriers to providing quality emergency obstetric care in Addis Ababa, Ethiopia: healthcare providers' perspectives on training, referrals and supervision, a mixed methods study. *BMC pregnancy and childbirth* 2015; **15**: 1–10.

29 Rowe AK, Rowe SY, Peters DH, Holloway KA, Chalker J, Ross-Degnan D. Effectiveness of strategies to improve health-care provider practices in low-income and middle-income countries: a systematic review. *The Lancet Global Health* 2018; **6**: e1163–75.

30 Garcia-Elorrio E, Rowe SY, Teijeiro ME, Ciapponi A, Rowe AK. The effectiveness of the quality improvement collaborative strategy in low- and middle-income countries: A systematic review and meta-analysis. *PloS one* 2019; **14**: e0221919.



For peer review only - http://bmjopen.bmj.com/site/about/guidelines.xhtml

31 Taneja G, Sarin E, Bajpayee D, *et al.* Care Around Birth Approach: A Training, Mentoring, and Quality Improvement Model to Optimize Intrapartum and Immediate Postpartum Quality of Care in India. *Global Health: Science and Practice* 2021; **9**: 590–610.

32 Nimako K, Kruk ME. Seizing the moment to rethink health systems. *The Lancet Global Health* 2021.

33 Kruk ME, Gage AD, Arsenault C, *et al.* High-quality health systems in the Sustainable Development Goals era: time for a revolution. *The Lancet Global Health* 2018; **6**: e1196–252.

For peer review only

**Figure 1**. Quality of intrapartum and immediate postpartum care processes by number of providers and facility type

**Figure 2**. Predicted quality of care by coworker performance and index provider performance
Notes: Points show the predicted estimate of quality by their coworkers' performance for the given level of index provider performance; bars show 95% confidence intervals. Gray line shows median performance.

For peer review only

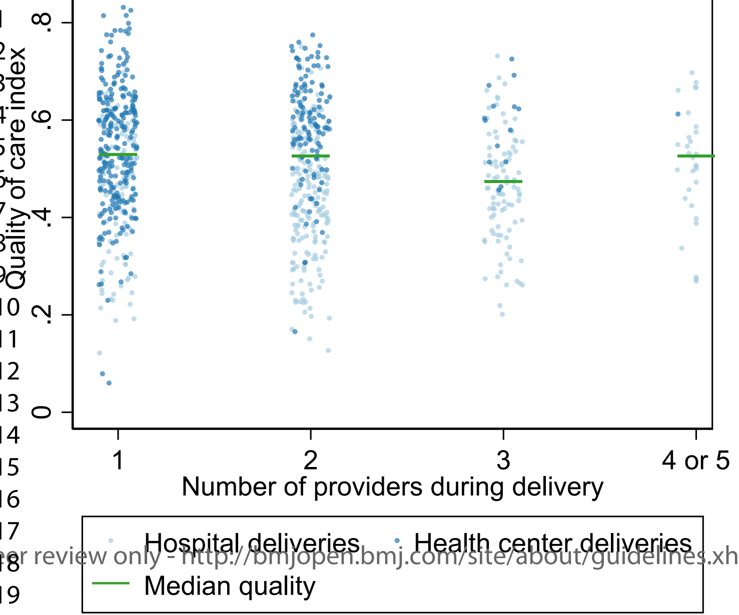


peer review only - http://bmjopen.bmj.com/site/about/guidelines.xh

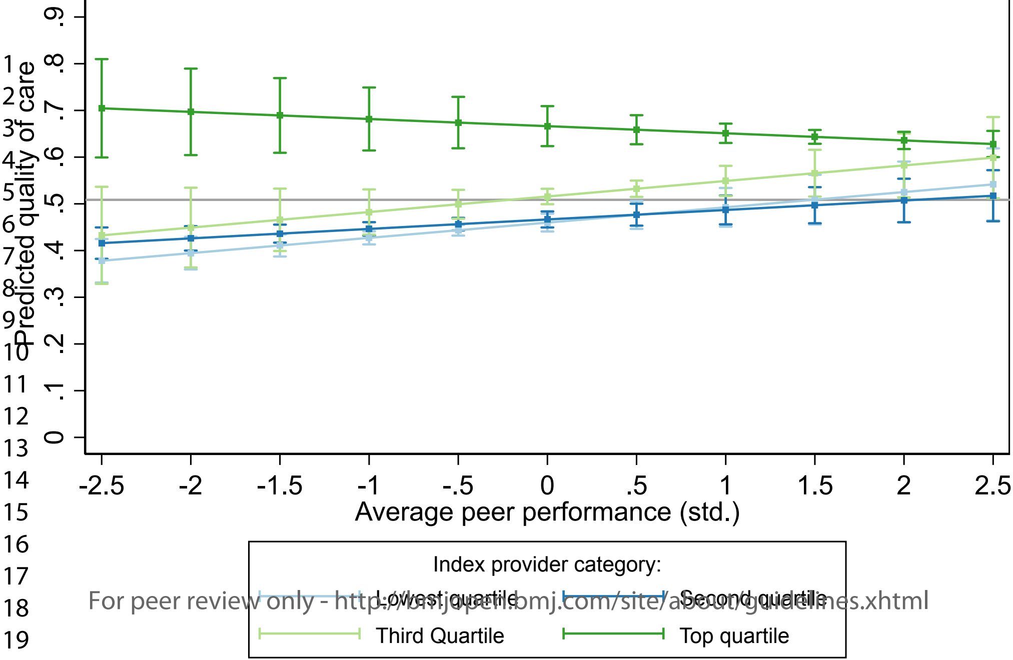

Predicted quality of care
0
.1
.2
.3
.4
.5
.6
.7
.8
.9
-2.5
-2
-1.5
-1
-.5
0
.5
1
1.5
2
2.5
Average peer performance (std.)
Index provider category:
Lowest quartile
Second quartile
Third Quartile
Top quartile

**Appendix 1.** Examples of provider arrangements during labor and delivery

| Number of providers | First exam | First stage of labor | Delivery care | Immediate newborn care | Immediate maternal postpartum care | Frequency |
|---|---|---|---|---|---|---|
| 1 | | | | | | 45% |
| 2 | | | | | | 38% |
| 3 | | | | | | 13% |
| 4 | | | | | | 3% |
| 5 | | | | | | 0.5% |

| Legend |
|---|
| Provider 1 |
| Provider 2 |
| Provider 3 |
| Provider 4 |
| Provider 5 |

For peer review only

**Appendix 2.** Quality of intrapartum and immediate postpartum care processes with no time constraints on actions

| | Percent completed | N |
|---|---|---|
| Average of first exam actions | 47% | 823 |
| Checks woman's HIV status | 68% | 809 |
| Asks whether woman has experienced headaches or blurred vision | 6% | 823 |
| Asks whether woman has experienced vaginal bleeding | 7% | 823 |
| Takes blood pressure during initial client assessment | 73% | 822 |
| Takes pulse during initial client assessment | 67% | 822 |
| Washes hands before initial examination | 13% | 823 |
| Wears gloves before vaginal examination | 99% | 781 |
| Average of first stage of labor actions | 48% | 795 |
| At least once, explains what will happen in labor | 42% | 824 |
| Prepares uterotonic drug to use for AMTSL | 81% | 791 |
| Uses partograph during labor | 53% | 793 |
| Prepares bags and masks for neonatal resuscitation | 14% | 783 |
| Average of third stage of labor actions | 88% | 828 |
| Correctly administers uterotonic (no time requirement) | 93% | 820 |
| Assesses completeness of placenta and membranes | 74% | 819 |
| Assesses for perineal and vaginal membranes | 90% | 819 |
| Ties or clamps cord | 99% | 828 |
| Average of immediate newborn care | 81% | 694 |
| Immediately dries baby with towel | 98% | 694 |
| Places newborn on mother's abdomen skin-to-skin | 64% | 690 |
| Average of immediate maternal postpartum stage actions | 37% | 824 |
| Takes mother's vital signs after birth | 3% | 823 |
| Palpates uterus after birth | 72% | 824 |
| Assists mother to initiate breastfeeding | 37% | 695 |

| | | |
|---|---|---|
| Alternate Quality of Intrapartum and Immediate Postpartum Care Processes index | 58% | 828 |

**Appendix 3**. Decomposition of variance in quality of care

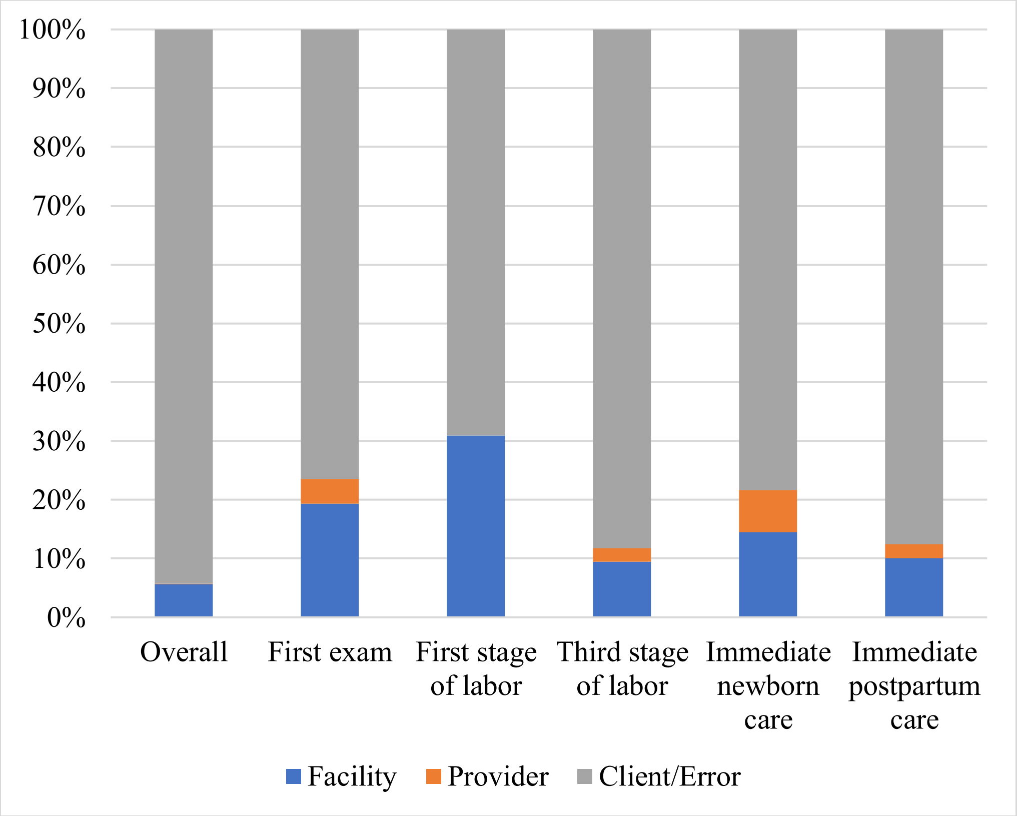


**Appendix 4.** Quality of care by number of providers

| | Quality of care | | |
|---|---|---|---|
| Number of providers | Mean | N observations | p-value |
| 1 | 0.518 | 371 | |
| 2 | 0.506 | 317 | 0.228 |
| 3 | 0.473 | 109 | 0.002 |
| 4 | 0.500 | 27 | 0.490 |
| 5 | 0.557 | 4 | 0.553 |
| Total | 0.507 | 828 | |

Notes: p-value for difference relative to solo deliveries (1 provider)

ew only

**Appendix 5.** Predictors of having multiple providers during a delivery

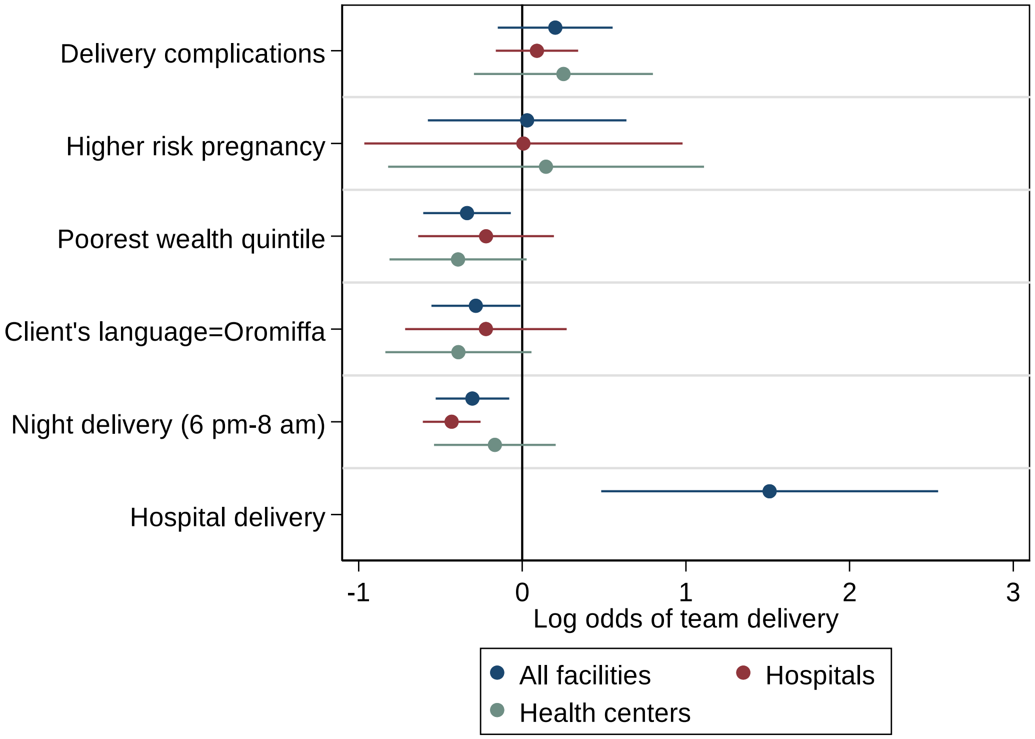


Notes: Points and confidence intervals show the log odds of predictors association with having more than one provider attend the delivery, with observations at the delivery level. Delivery complication is neonatal resuscitation, newborn referred to the NICU, or mother treated for post-partum hemorrhage or eclampsia. Higher risk pregnancy is mother grand multiparous (5 or more births), younger than 18 or older than 35, or has multiple births.

**Appendix 6.** Bivariate relationships between independent variables and quality of care

| | Coef. | p value | 95% CI | N |
|---|---|---|---|---|
| Coworkers' performance across all deliveries | 0.059 | 0.00 | (0.04,0.07) | 2189 |
| Index provider is most senior in group | -0.056 | 0.01 | (-0.1,-0.02) | 2189 |
| Index provider is most experienced in group | 0.003 | 0.82 | (-0.02,0.03) | 2189 |
| Number of providers | -0.006 | 0.53 | (-0.03,0.01) | 2189 |
| Years of experience | 0.003 | 0.44 | (0,0.01) | 2101 |
| Delivery complication | -0.002 | 0.92 | (-0.04,0.04) | 2189 |
| Higher risk pregnancy | -0.001 | 0.97 | (-0.04,0.04) | 2186 |
| Night delivery | 0.012 | 0.40 | (-0.02,0.04) | 2189 |
| Provider cadre (Midwife or nurse diploma ref.) | | | | |
| Midwife or nurse bsc. | 0.047 | 0.09 | (-0.01,0.1) | 2149 |
| Health officer | -0.052 | 0.17 | (-0.13,0.02) | 2149 |
| GP | -0.037 | 0.14 | (-0.09,0.01) | 2149 |
| IESO | 0.024 | 0.85 | (-0.22,0.27) | 2149 |
| Client wealth index (poorest reference) | | | | |
| Wealth 2 | 0.043 | 0.05 | (0,0.09) | 1990 |
| Wealth 3 | 0.077 | 0.00 | (0.03,0.12) | 1990 |
| Wealth 4 | 0.026 | 0.18 | (-0.01,0.06) | 1990 |
| Wealth 5 (wealthiest) | 0.039 | 0.05 | (0,0.08) | 1990 |
| Delivery stage (first exam ref.) | | | | |
| First stage of labor | -0.001 | 0.96 | (-0.04,0.03) | 2189 |
| Third stage of labor | 0.228 | 0.00 | (0.2,0.26) | 2189 |
| Immediate newborn care | 0.294 | 0.00 | (0.25,0.33) | 2189 |
| Immediate maternal postpartum care | -0.323 | 0.00 | (-0.36,-0.29) | 2189 |
| Facility (Dil Chorra Hospital ref.) | | | | |
| Sabien Primary Hospital | 0.028 | 0.06 | (0,0.06) | 2189 |
| Biyowale Health Center | 0.067 | 0.20 | (-0.04,0.17) | 2189 |
| Legeharae Health Center | 0.189 | 0.00 | (0.15,0.23) | 2189 |
| Melka Jebdu Health Center | 0.139 | 0.00 | (0.09,0.19) | 2189 |
| Wahil Health Center | -0.020 | 0.10 | (-0.04,0) | 2189 |
| Gende Gerada Health Center | 0.034 | 0.31 | (-0.03,0.1) | 2189 |
| Goro Health Center | 0.197 | 0.00 | (0.16,0.23) | 2189 |
| Jelobelina Health Center | 0.189 | 0.00 | (0.12,0.26) | 2189 |

For peer review only

**Appendix 7.** Index provider and coworker provider performance among 2 provider deliveries (N=1326)

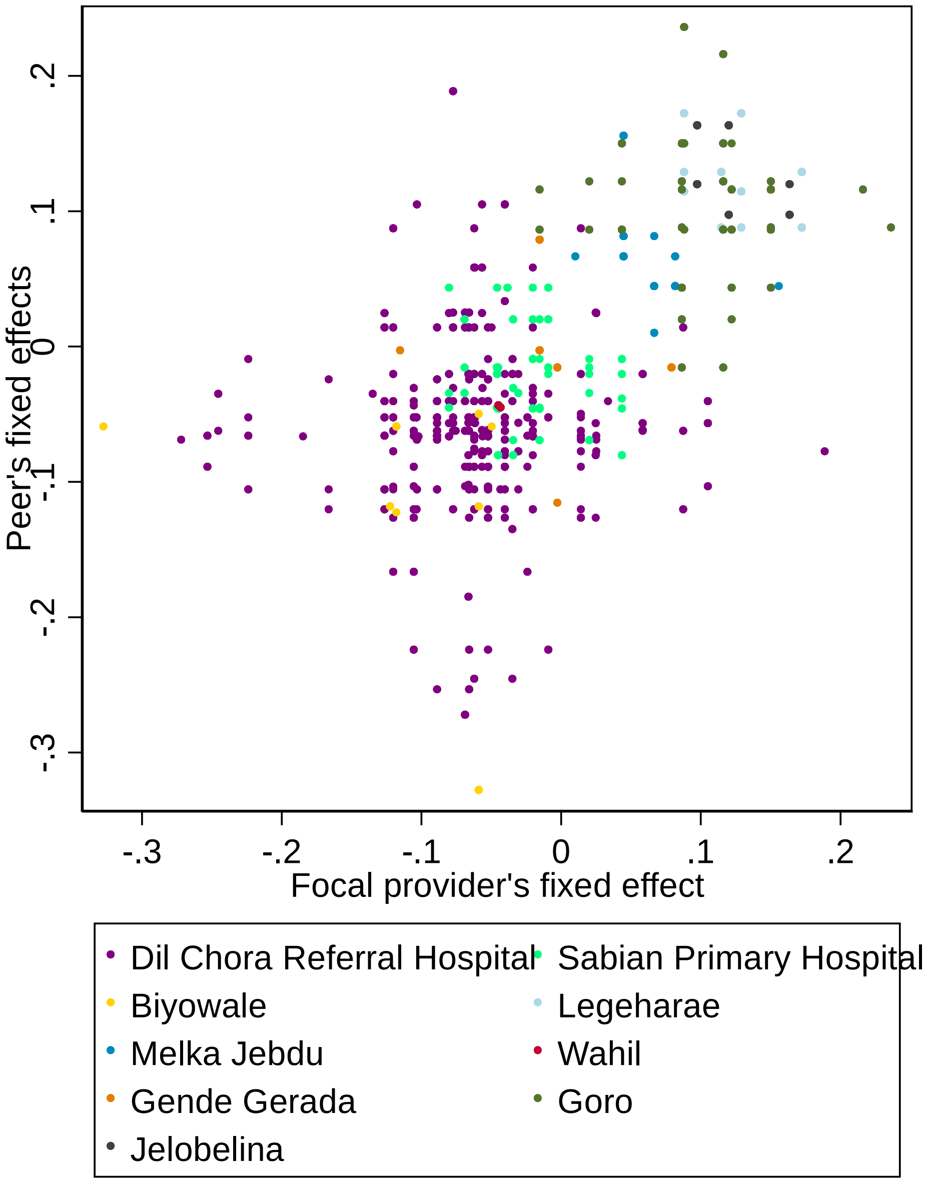


Index provider and coworker provider performance among deliveries with >2 providers (N=674)

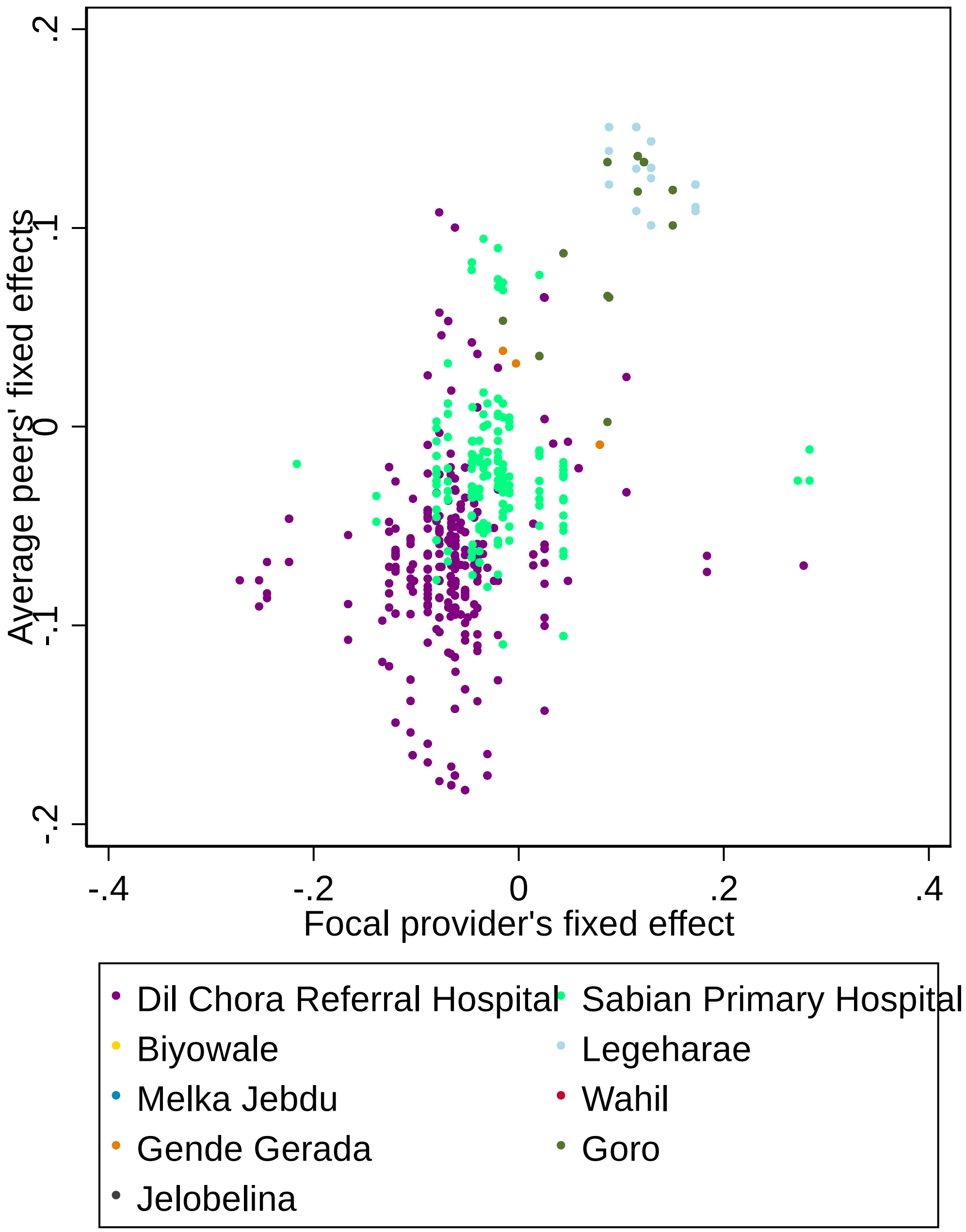
Average peers' fixed effects
.2
.1
0
-.1
-.2
-.4
-.2
0
.2
.4
Focal provider's fixed effect
Dil Chora Referral Hospital
Sabian Primary Hospital
Biyowale
Legeharae
Melka Jebdu
Wahil
Gende Gerada
Goro
Jelobelina

**Appendix 8.** Multivariable associations between group characteristics and quality of care by delivery stage

| | First exam | | First stage of labor | | Third stage of labor | | Immediate newborn | | Immediate maternal postpartum | |
|---|---|---|---|---|---|---|---|---|---|---|
| | Coef. | p value | Coef. | p value | Coef. | p value | Coef. | p value | Coef. | p value |
| Group characteristics | | | | | | | | | | |
| Peers' performance across all deliveries | -0.005 | 0.77 | 0.042 | 0.01 | 0.020 | 0.10 | 0.036 | 0.09 | 0.023 | 0.15 |
| Peers are more senior cadre than index | -0.016 | 0.65 | -0.097 | 0.00 | -0.041 | 0.13 | -0.059 | 0.21 | 0.004 | 0.90 |
| Peers are more experienced than index | 0.000 | 0.98 | -0.002 | 0.96 | -0.011 | 0.56 | 0.000 | 0.99 | -0.030 | 0.12 |
| Number of providers | -0.011 | 0.42 | 0.039 | 0.05 | -0.002 | 0.89 | -0.021 | 0.34 | -0.007 | 0.61 |
| Index provider characteristics | | | | | | | | | | |
| Years of experience | -0.001 | 0.75 | 0.000 | 0.92 | 0.000 | 0.90 | 0.000 | 0.98 | -0.003 | 0.35 |
| Provider cadre (Midwife or nurse diploma ref.) | | | | | | | | | | |
| Midwife or nurse Bsc. | -0.065 | 0.05 | -0.140 | 0.00 | -0.034 | 0.20 | -0.073 | 0.16 | 0.065 | 0.03 |
| Health officer | -0.142 | 0.08 | -0.016 | 0.82 | -0.161 | 0.12 | -0.506 | 0.06 | 0.043 | 0.72 |
| GP | -0.002 | 0.97 | -0.135 | 0.00 | -0.053 | 0.13 | -0.176 | 0.00 | -0.025 | 0.52 |
| IESO | -0.206 | 0.05 | -0.088 | 0.57 | 0.035 | 0.77 | -0.273 | 0.09 | -0.071 | 0.09 |
| Context and environment | | | | | | | | | | |
| Delivery complication | 0.044 | 0.02 | -0.039 | 0.24 | 0.012 | 0.66 | 0.030 | 0.34 | -0.005 | 0.87 |
| Higher risk pregnancy | -0.022 | 0.52 | 0.038 | 0.28 | 0.013 | 0.65 | 0.003 | 0.96 | -0.006 | 0.86 |
| Night delivery (morning reference) | 0.006 | 0.75 | 0.002 | 0.93 | -0.021 | 0.22 | 0.021 | 0.36 | 0.027 | 0.22 |
| Client wealth index (poorest reference) | | | | | | | | | | |
| Wealth 2 | 0.013 | 0.67 | 0.095 | 0.02 | -0.004 | 0.88 | 0.040 | 0.31 | 0.015 | 0.60 |
| Wealth 3 | 0.038 | 0.22 | 0.123 | 0.00 | 0.066 | 0.05 | 0.110 | 0.02 | 0.035 | 0.30 |
| Wealth 4 | 0.014 | 0.63 | 0.066 | 0.11 | 0.030 | 0.37 | 0.039 | 0.37 | 0.043 | 0.20 |
| Wealth 5 (wealthiest) | 0.002 | 0.94 | 0.060 | 0.11 | 0.046 | 0.10 | 0.084 | 0.06 | 0.053 | 0.06 |
| Facility (Dil Chorra Hospital ref.) | | | | | | | | | | |
| Sabien Primary Hospital | 0.156 | 0.00 | 0.013 | 0.62 | -0.022 | 0.51 | -0.041 | 0.42 | -0.034 | 0.29 |

For peer review only

| | | | | | | | | | | |
|---|---|---|---|---|---|---|---|---|---|---|
| Biyowale Health Center | -0.150 | 0.00 | 0.122 | 0.36 | 0.132 | 0.01 | 0.335 | 0.00 | 0.171 | 0.23 |
| Legeharae Health Center | 0.114 | 0.01 | 0.188 | 0.00 | 0.116 | 0.00 | 0.129 | 0.06 | 0.122 | 0.01 |
| Melka Jebdu Health Center | 0.104 | 0.08 | 0.173 | 0.02 | 0.106 | 0.00 | 0.089 | 0.11 | 0.101 | 0.15 |
| Wahil Health Center | 0.078 | 0.09 | -0.157 | 0.00 | -0.002 | 0.94 | -0.272 | 0.00 | 0.109 | 0.00 |
| Gende Gerada Health Center | 0.085 | 0.16 | 0.122 | 0.00 | 0.076 | 0.24 | 0.217 | 0.23 | 0.004 | 0.97 |
| Goro Health Center | 0.152 | 0.00 | 0.344 | 0.00 | 0.050 | 0.15 | 0.178 | 0.01 | -0.003 | 0.96 |
| Jelobelina Health Center | 0.133 | 0.07 | 0.243 | 0.00 | 0.158 | 0.01 | 0.160 | 0.05 | 0.106 | 0.04 |
| Constant | 0.470 | 0.00 | 0.380 | 0.00 | 0.729 | 0.00 | 0.778 | 0.00 | 0.063 | 0.34 |
| N obs | 454 | | 440 | | 453 | | 387 | | 455 | |

For peer review only

**Appendix 9.** Comparison of average vs best coworker performance for deliveries with 3 or more providers

| | Coworkers' average performance | | Best coworker's performance | |
|---|---|---|---|---|
| | Coef. | p value | Coef. | p value |
| Group characteristics | | | | |
| Coworkers' average performance across all deliveries | 0.045 | 0.01 | | |
| Best coworker's performance across all deliveries | | | 0.023 | 0.08 |
| Coworkers are more senior cadre than index | -0.054 | 0.04 | -0.062 | 0.02 |
| Coworkers are more experienced than index | -0.024 | 0.27 | -0.023 | 0.29 |
| Number of providers | 0.008 | 0.68 | 0.003 | 0.88 |
| Index provider characteristics | | | | |
| Years of experience | -0.005 | 0.25 | -0.004 | 0.29 |
| Provider cadre (Midwife or nurse diploma ref.) | | | | |
| Midwife or nurse Bsc. | -0.053 | 0.05 | -0.06 | 0.04 |
| Health officer | -0.173 | 0.34 | -0.181 | 0.33 |
| GP | -0.088 | 0.02 | -0.093 | 0.02 |
| IESO | 0.001 | 0.99 | -0.004 | 0.97 |
| Context and environment | | | | |
| Delivery complication | 0.015 | 0.52 | 0.017 | 0.47 |
| Higher risk pregnancy | 0.018 | 0.57 | 0.018 | 0.58 |
| Night delivery (morning reference) | 0.052 | 0.01 | 0.052 | 0.01 |
| Client wealth index (poorest reference) | | | | |
| Wealth 2 | -0.028 | 0.40 | -0.03 | 0.37 |
| Wealth 3 | 0.022 | 0.47 | 0.017 | 0.56 |
| Wealth 4 | -0.019 | 0.57 | -0.023 | 0.50 |
| Wealth 5 (wealthiest) | -0.015 | 0.63 | -0.019 | 0.55 |
| Delivery stage (first exam ref.) | | | | |
| First stage of labor | -0.01 | 0.71 | -0.011 | 0.68 |
| Third stage of labor | 0.208 | 0.00 | 0.208 | 0.00 |
| Immediate newborn care | 0.247 | 0.00 | 0.246 | 0.00 |
| Immediate postpartum care | -0.335 | 0.00 | -0.335 | 0.00 |
| Facility (Dil Chorra Hospital ref.) | | | | |
| Sabien Primary Hospital | 0.034 | 0.23 | 0.044 | 0.12 |
| Legeharae Health Center | 0.052 | 0.45 | 0.102 | 0.11 |
| Gende Gerada Health Center | 0.139 | 0.16 | 0.162 | 0.10 |
| Goro Health Center | 0.1 | 0.09 | 0.141 | 0.01 |
| Constant | 0.453 | 0.00 | 0.46 | 0.00 |
| N | 615 | | 615 | |

For peer review only

**Appendix 10.** Association between coworker performance and quality by provider cadre rank

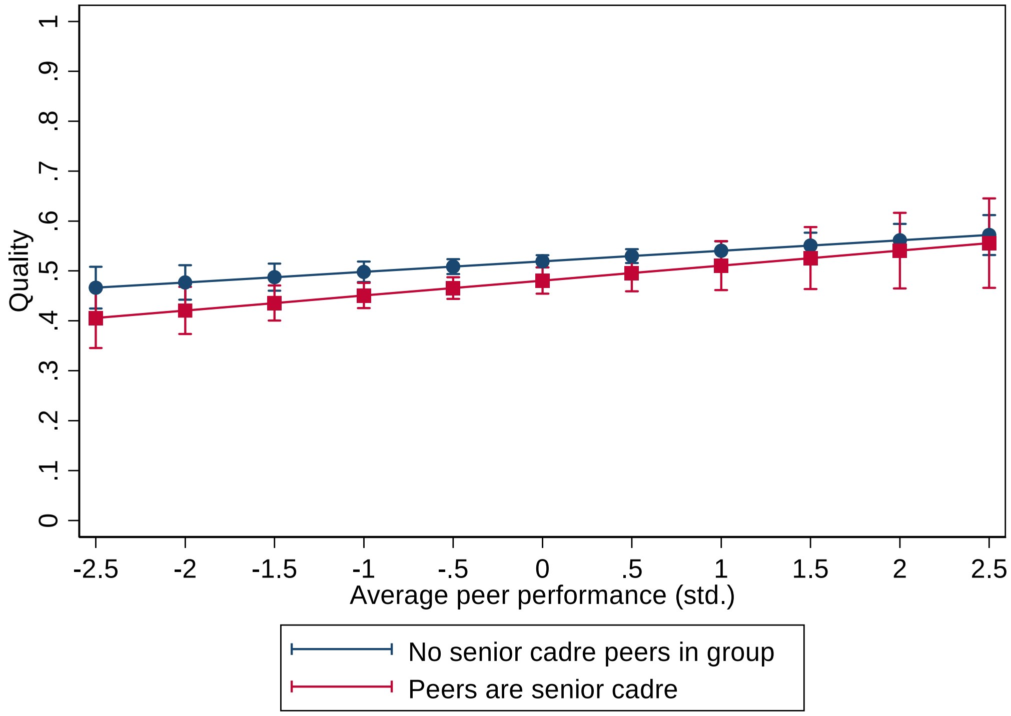


**Appendix 11**. Association between coworker performance and quality by provider seniority (years of experience)

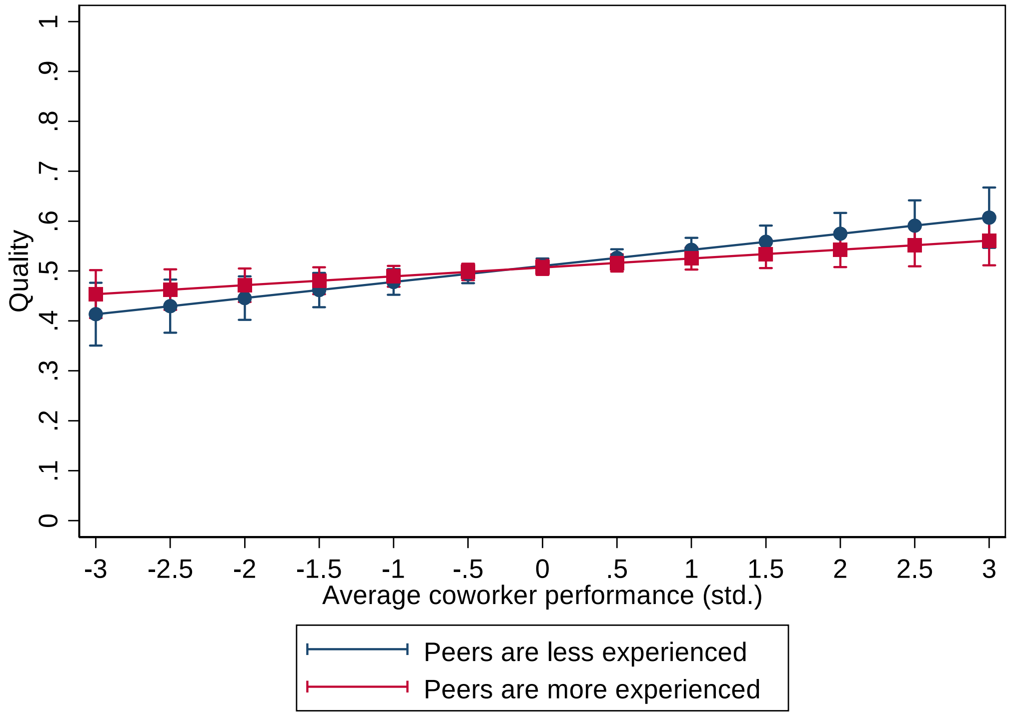

STROBE Statement—Checklist of items that should be included in reports of ***cross-sectional studies***

| | Item No | Recommendation | Page No |
|---|---|---|---|
| **Title and abstract** | 1 | (*a*) Indicate the study's design with a commonly used term in the title or the abstract | 1 |
| | | (*b*) Provide in the abstract an informative and balanced summary of what was done and what was found | 1 |
| **Introduction** | | | |
| Background/rationale | 2 | Explain the scientific background and rationale for the investigation being reported | 2 |
| Objectives | 3 | State specific objectives, including any prespecified hypotheses | 2 |
| **Methods** | | | |
| Study design | 4 | Present key elements of study design early in the paper | 2 |
| Setting | 5 | Describe the setting, locations, and relevant dates, including periods of recruitment, exposure, follow-up, and data collection | 3,4 |
| Participants | 6 | (*a*) Give the eligibility criteria, and the sources and methods of selection of participants | 4 |
| Variables | 7 | Clearly define all outcomes, exposures, predictors, potential confounders, and effect modifiers. Give diagnostic criteria, if applicable | 4-7 |
| Data sources/ measurement | 8* | For each variable of interest, give sources of data and details of methods of assessment (measurement). Describe comparability of assessment methods if there is more than one group | *4-7* |
| Bias | 9 | Describe any efforts to address potential sources of bias | 7 |
| Study size | 10 | Explain how the study size was arrived at | |
| Quantitative variables | 11 | Explain how quantitative variables were handled in the analyses. If applicable, describe which groupings were chosen and why | 4-7 |
| Statistical methods | 12 | (*a*) Describe all statistical methods, including those used to control for confounding | 7 |
| | | (*b*) Describe any methods used to examine subgroups and interactions | 7 |
| | | (*c*) Explain how missing data were addressed | 7 |
| | | (*d*) If applicable, describe analytical methods taking account of sampling strategy | 7 |
| | | (*e*) Describe any sensitivity analyses | 5,7 |
| **Results** | | | |
| Participants | 13* | (a) Report numbers of individuals at each stage of study—eg numbers potentially eligible, examined for eligibility, confirmed eligible, included in the study, completing follow-up, and analysed | 7 |
| | | (b) Give reasons for non-participation at each stage | 7 |
| | | (c) Consider use of a flow diagram | |
| Descriptive data | 14* | (a) Give characteristics of study participants (eg demographic, clinical, social) and information on exposures and potential confounders | Table 2 |
| | | (b) Indicate number of participants with missing data for each variable of interest | Table 2 |



For peer review only - http://bmjopen.bmj.com/site/about/guidelines.xhtml

| | | | |
|---|---|---|---|
| Outcome data | 15* | Report numbers of outcome events or summary measures | Table 1 and Figure 1 |
| Main results | 16 | (*a*) Give unadjusted estimates and, if applicable, confounder-adjusted estimates and their precision (eg, 95% confidence interval). Make clear which confounders were adjusted for and why they were included | Table 3 and Appendix |
| | | (*b*) Report category boundaries when continuous variables were categorized | N/A |
| | | (*c*) If relevant, consider translating estimates of relative risk into absolute risk for a meaningful time period | 9 |
| Other analyses | 17 | Report other analyses done—eg analyses of subgroups and interactions, and sensitivity analyses | Figure 2 |
| **Discussion** | | | |
| Key results | 18 | Summarise key results with reference to study objectives | 11 |
| Limitations | 19 | Discuss limitations of the study, taking into account sources of potential bias or imprecision. Discuss both direction and magnitude of any potential bias | 12 |
| Interpretation | 20 | Give a cautious overall interpretation of results considering objectives, limitations, multiplicity of analyses, results from similar studies, and other relevant evidence | 11 |
| Generalisability | 21 | Discuss the generalisability (external validity) of the study results | 12 |
| **Other information** | | | |
| Funding | 22 | Give the source of funding and the role of the funders for the present study and, if applicable, for the original study on which the present article is based | submission |

*Give information separately for exposed and unexposed groups.

**Note:** An Explanation and Elaboration article discusses each checklist item and gives methodological background and published examples of transparent reporting. The STROBE checklist is best used in conjunction with this article (freely available on the Web sites of PLoS Medicine at http://www.plosmedicine.org/, Annals of Internal Medicine at http://www.annals.org/, and Epidemiology at http://www.epidem.com/). Information on the STROBE Initiative is available at www.strobe-statement.org.

For peer review only


For peer review only - http://bmjopen.bmj.com/site/about/guidelines.xhtml
